# Supplementary material for: IP3 receptor depletion in a spontaneous canine model of Charcot-Marie-Tooth disease 1J with amelogenesis imperfecta
Source: PLoS Genet. 2025 Jan 13;21(1):e1011328. doi: 10.1371/journal.pgen.1011328 (PMC11761660; doi:10.1371/journal.pgen.1011328)
Supplement: S1 Fig — (PDF) [file pgen.1011328.s001.pdf]

Nonsense variant in ITPR3 leads to depletion of IP3 receptors in dogs with developmental dental defect and decreased nerve conduction velocity

Marjo K. Hytönen[#], Julius Rönkkö[#], Sruthi Hundi, Tarja S. Jokinen, Emilia Suonto, Eeva Teräväinen, Jonas Donner, Rita La Rovere, Geert Bultynck, Emil Ylikallio, Henna Tyynismaa and Hannes Lohi*

*Corresponding author: Hannes Lohi (hannes.lohi@helsinki.fi), Department of Veterinary Biosciences, Department of Medical and Clinical Genetics, Folkhälsan Research Center, University of Helsinki, Finland.

Percent identity matrices (Clustal 2.1).

ITPR1	Canine	Human	Mouse	Rat
Canine	100	98.73	98.69	98.62
Human	98.73	100	98.62	98.51
Mouse	98.69	98.62	100	99.6
Rat	98.62	98.51	99.6	100

ITPR2	Canine	Human	Mouse	Rat
Canine	100	97.93	95.48	95.3
Human	97.93	100	95.82	95.34
Mouse	95.48	95.82	100	98.41
Rat	95.3	95.34	98.41	100

ITPR3	Canine	Human	Mouse	Rat
Canine	100	96.41	94.27	94.23
Human	96.41	100	95.32	95.24
Mouse	94.27	95.32	100	98.73
Rat	94.23	95.24	98.73	100

Alignment of the dog ITPR1 protein sequence (A0A8I3NKB2) to human (Q14643), mouse (P11881) and rat (P29994) ITPR1 with the Clustal Omega algorithm (CLUSTAL O(1.2.4)).

```
sp|P11881|ITPR1_MOUSE              MSDKMSSFLHIGDICSLYAEGSTNGFISTLGLVDDRCVVQPEAGDLNNPPKKFRDCLFKL         60
sp|P29994|ITPR1_RAT                MSDKMSSFLHIGDICSLYAEGSTNGFISTLGLVDDRCVVQPEAGDLNNPPKKFRDCLFKL         60
sp|Q14643|ITPR1_HUMAN              MSDKMSSFLHIGDICSLYAEGSTNGFISTLGLVDDRCVVQPETGDLNNPPKKFRDCLFKL         60
tr|A0A8I3NKB2|A0A8I3NKB2_CANLF     MSDKMSSFLHIGDICSLYAEGSTNGFISTLGLVDDRCVVQPEAGDLNNPPKKFRDCLFKL         60
                                   ******************************************:*****************

sp|P11881|ITPR1_MOUSE              CPMNRYSAQKQFWKAAKPGANSTTDAVLLNKLHHAADLEKKQNETENRKLLGTVIQYGNV         120
sp|P29994|ITPR1_RAT                CPMNRYSAQKQFWKAAKPGANSTTDAVLLNKLHHAADLEKKQNETENRKLLGTVIQYGNV         120
sp|Q14643|ITPR1_HUMAN              CPMNRYSAQKQFWKAAKPGANSTTDAVLLNKLHHAADLEKKQNETENRKLLGTVIQYGNV         120
tr|A0A8I3NKB2|A0A8I3NKB2_CANLF     CPMNRYSAQKQFWKAAKPGANSTTDAVLLNKLHHAADLEKKQNETENRKLLGTVIQYGNV         120
                                   ************************************************************

sp|P11881|ITPR1_MOUSE              IQLLHLKSNKYLTVNKRLPALLEKNAMRVTLDEAGNEGSWFYIQPFYKLRSIGDSVVIGD         180
sp|P29994|ITPR1_RAT                IQLLHLKSNKYLTVNKRLPALLEKNAMRVTLDEAGNEGSWFYIQPFYKLRSIGDSVVIGD         180
sp|Q14643|ITPR1_HUMAN              IQLLHLKSNKYLTVNKRLPALLEKNAMRVTLDEAGNEGSWFYIQPFYKLRSIGDSVVIGD         180
tr|A0A8I3NKB2|A0A8I3NKB2_CANLF     IQLLHLKSNKYLTVNKRLPALLEKNAMRVTLDEAGNEGSWFYIQPFYKLRSIGDSVVIGD         180
                                   ************************************************************

sp|P11881|ITPR1_MOUSE              KVVLNPVNAGQPLHASSHQLVDNPGCNEVNSVNCNTSWKIVLFMKWSDNKDDILKGGDVV         240
sp|P29994|ITPR1_RAT                KVVLNPVNAGQPLHASSHQLVDNPGCNEVNSVNCNTSWKIVLFMKWSDNKDDILKGGDVV         240
sp|Q14643|ITPR1_HUMAN              KVVLNPVNAGQPLHASSHQLVDNPGCNEVNSVNCNTSWKIVLFMKWSDNKDDILKGGDVV         240
tr|A0A8I3NKB2|A0A8I3NKB2_CANLF     KVVLNPVNAGQPLHASSHQLVDNPGCNEVNSVNCNTSWKIVLFMKWSDNKDDILKGGDVV         240
                                   ************************************************************

sp|P11881|ITPR1_MOUSE              RLFHAEQEKFLTCDEHRKKQHVFLRTTGRQSATSATSSKALWEVEVVQHDPCRGGAGYWN         300
sp|P29994|ITPR1_RAT                RLFHAEQEKFLTCDEHRKKQHVFLRTTGRQSATSATSSKALWEVEVVQHDPCRGGAGYWN         300
sp|Q14643|ITPR1_HUMAN              RLFHAEQEKFLTCDEHRKKQHVFLRTTGRQSATSATSSKALWEVEVVQHDPCRGGAGYWN         300
tr|A0A8I3NKB2|A0A8I3NKB2_CANLF     RLFHAEQEKFLTCDEHRKKQHVFLRTTGRQSATSATSSKALWEVEVVQHDPCRGGAGYWN         300
                                   ************************************************************

sp|P11881|ITPR1_MOUSE              SLFRFKHLATGHYLAAEVDPDFEEECLEFQPSVDPDQDASRSRLRNAQEKMVYSLVSVPE         360
sp|P29994|ITPR1_RAT                SLFRFKHLATGHYLAAEVDPDFEEECLEFQPSVDPDQDASRSRLRNAQEKMVYSLVSVPE         360
sp|Q14643|ITPR1_HUMAN              SLFRFKHLATGHYLAAEVDPDFEEECLEFQPSVDPDQDASRSRLRNAQEKMVYSLVSVPE         360
tr|A0A8I3NKB2|A0A8I3NKB2_CANLF     SLFRFKHLATGHYLAAEVDPDFEEECLEFQPSVDPDQDASRSRLRNAQEKMVYSLVSVPE         360
                                   ************************************************************

sp|P11881|ITPR1_MOUSE              GNDISSIFELDPTTLRGGDSLVPRNSYVRLRHLCTNTWVHSTNIPIDKEEEKPVMLKIGT         420
sp|P29994|ITPR1_RAT                GNDISSIFELDPTTLRGGDSLVPRNSYVRLRHLCTNTWVHSTNIPIDKEEEKPVMLKIGT         420
sp|Q14643|ITPR1_HUMAN              GNDISSIFELDPTTLRGGDSLVPRNSYVRLRHLCTNTWVHSTNIPIDKEEEKPVMLKIGT         420
tr|A0A8I3NKB2|A0A8I3NKB2_CANLF     GNDISSIFELDPTTLRGGDSLVPRNSYVRLRHLCTNTWVHSTNIPIDKEEEKPVMLKIGT         420
                                   ************************************************************
```

```
sp|P11881|ITPR1_MOUSE              SPLKEDKEAFAIVPVSPAEVRDLDFANDASKVLGSIAGKLEKGTITQNERRSVTKLLEDL      480
sp|P29994|ITPR1_RAT                SPLKEDKEAFAIVPVSPAEVRDLDFANDASKVLGSIAGKLEKGTITQNERRSVTKLLEDL      480
sp|Q14643|ITPR1_HUMAN              SPVKEDKEAFAIVPVSPAEVRDLDFANDASKVLGSIAGKLEKGTITQNERRSVTKLLEDL      480
tr|A0A8I3NKB2|A0A8I3NKB2_CANLF     SPVKEDKEAFAIVPVSPAEVRDLDFANDASKVLGSIAGKLEKGTITQNERRSVTKLLEDL      480
                                   **:*********************************************************

sp|P11881|ITPR1_MOUSE              VYFVTGGTNSGQDVLEVVFSKPNRERQKLMREQNILKQIFKLLQAPFTDCGDGPMLRLEE      540
sp|P29994|ITPR1_RAT                VYFVTGGTNSGQDVLEVVFSKPNRERQKLMREQNILKQIFKLLQAPFTDCGDGPMLRLEE      540
sp|Q14643|ITPR1_HUMAN              VYFVTGGTNSGQDVLEVVFSKPNRERQKLMREQNILKQIFKLLQAPFTDCGDGPMLRLEE      540
tr|A0A8I3NKB2|A0A8I3NKB2_CANLF     VYFVTGGTNSGQDVLEVVFSKPNRERQKLMREQNILKQIFKLLQAPFTDCGDGPMLRLEE      540
                                   ************************************************************

sp|P11881|ITPR1_MOUSE              LGDQRHAPFRHICRLCYRVLRHSQQDYRKNQEYIAKQFGFMQKQIGYDVLAEDTITALLH      600
sp|P29994|ITPR1_RAT                LGDQRHAPFRHICRLCYRVLRHSQQDYRKNQEYIAKQFGFMQKQIGYDVLAEDTITALLH      600
sp|Q14643|ITPR1_HUMAN              LGDQRHAPFRHICRLCYRVLRHSQQDYRKNQEYIAKQFGFMQKQIGYDVLAEDTITALLH      600
tr|A0A8I3NKB2|A0A8I3NKB2_CANLF     LGDQRHAPFRHICRLCYRVLRHSQQDYRKNQEYIAKQFGFMQKQIGYDVLAEDTITALLH      600
                                   ************************************************************

sp|P11881|ITPR1_MOUSE              NNRKLLEKHITAAEIDTFVSLVRKNREPRFLDYLSDLCVSMNKSIPVTQELICKAVLNPT      660
sp|P29994|ITPR1_RAT                NNRKLLEKHITAAEIDTFVSLVRKNREPRFLDYLSDLCVSMNKSIPVTQELICKAVLNPT      660
sp|Q14643|ITPR1_HUMAN              NNRKLLEKHITAAEIDTFVSLVRKNREPRFLDYLSDLCVSMNKSIPVTQELICKAVLNPT      660
tr|A0A8I3NKB2|A0A8I3NKB2_CANLF     NNRKLLEKHITAAEIDTFVSLVRKNREPRFLDYLSDLCVSMNKSIPVTQELICKAVLNPT      660
                                   ************************************************************

sp|P11881|ITPR1_MOUSE              NADILIETKLVLSRFEFEGVS-TGENALEAGEDEEEVWLFWRDSNKEIRSKSVRELAQDA      719
sp|P29994|ITPR1_RAT                NADILIETKLVLSRFEFEGVS-TGENALEAGEDEEEVWLFWRDSNKEIRSKSVRELAQDA      719
sp|Q14643|ITPR1_HUMAN              NADILIETKLVLSRFEFEGVSSTGENALEAGEDEEEVWLFWRDSNKEIRSKSVRELAQDA      720
tr|A0A8I3NKB2|A0A8I3NKB2_CANLF     NADILIETKLVLSRFEFEGVS-TGENALEAGEDEEEVWLFWRDSNKEIRSKSVRELAQDA      719
                                   ********************* **************************************

sp|P11881|ITPR1_MOUSE              KEGQKEDRDILSYYRYQLNLFARMCLDRQYLAINEISGQLDVDLILRCMSDENLPYDLRA      779
sp|P29994|ITPR1_RAT                KEGQKEDRDVLSYYRYQLNLFARMCLDRQYLAINEISGQLDVDLILRCMSDENLPYDLRA      779
sp|Q14643|ITPR1_HUMAN              KEGQKEDRDVLSYYRYQLNLFARMCLDRQYLAINEISGQLDVDLILRCMSDENLPYDLRA      780
tr|A0A8I3NKB2|A0A8I3NKB2_CANLF     KEGQKEDRDVLSYYRYQLNLFARMCLDRQYLAINEISGQLDVDLILRCMSDENLPYDLRA      779
                                   *********:**************************************************

sp|P11881|ITPR1_MOUSE              SFCRLMLHMHVDRDPQEQVTPVKYARLWSEIPSEIAIDDYDSSGTSKDEIKERFAQTMEF      839
sp|P29994|ITPR1_RAT                SFCRLMLHMHVDRDPQEQVTPVKYARLWSEIPSEIAIDDYDSSGASKDEIKERFAQTMEF      839
sp|Q14643|ITPR1_HUMAN              SFCRLMLHMHVDRDPQEQVTPVKYARLWSEIPSEIAIDDYDSSGASKDEIKERFAQTMEF      840
tr|A0A8I3NKB2|A0A8I3NKB2_CANLF     SFCRLMLHMHVDRDPQEQVTPVKYARLWSEIPSEIAIDDYDSSGASKDEIKERFAQTMEF      839
                                   ********************************************:***************

sp|P11881|ITPR1_MOUSE              VEEYLRDVVCQRFPFSDKEKNKLTFEVVNLARNLIYFGFYNFSDLLRLTKILLAILDCVH      899
sp|P29994|ITPR1_RAT                VEEYLRDVVCQRFPFSDKEKNKLTFEVVNLARNLIYFGFYNFSDLLRLTKILLAILDCVH      899
sp|Q14643|ITPR1_HUMAN              VEEYLRDVVCQRFPFSDKEKNKLTFEVVNLARNLIYFGFYNFSDLLRLTKILLAILDCVH      900
tr|A0A8I3NKB2|A0A8I3NKB2_CANLF     VEEYLRDVVCQRFPFSDKEKNKLTFEVVNLARNLIYFGFYNFSDLLRLTKILLAILDCVH      899
                                   ************************************************************

sp|P11881|ITPR1_MOUSE              VTTIFPISKMTKGEENKG---------SNVMRSIHGVGELMTQVVLRGGGFLPMTPMAAA      950
sp|P29994|ITPR1_RAT                VTTIFPISKMTKGEENKG---------SNVMRSIHGVGELMTQVVLRGGGFLPMTPMAAA      950
sp|Q14643|ITPR1_HUMAN              VTTIFPISKMAKGEENKGNNDVEKLKSSNVMRSIHGVGELMTQVVLRGGGFLPMTPMAAA      960
tr|A0A8I3NKB2|A0A8I3NKB2_CANLF     VTTIFPISKMAKGEENKGNKNVVAKWRSSNVMRSIHGVGELMTQVVLRGGGFLPMTPMAAA      959
                                   **********:*******         *********************************

sp|P11881|ITPR1_MOUSE              PEGNVKQAEPEKEDIMVMDTKLKIIEILQFILNVRLDYRISCLLCIFKREFDESNSQSSE      1010
sp|P29994|ITPR1_RAT                PEGNVKQAEPEKEDIMVMDTKLKIIEILQFILNVRLDYRISCLLCIFKREFDESNSQSSE      1010
sp|Q14643|ITPR1_HUMAN              PEGNVKQAEPEKEDIMVMDTKLKIIEILQFILNVRLDYRISCLLCIFKREFDESNSQTSE      1020
tr|A0A8I3NKB2|A0A8I3NKB2_CANLF     PEGNVKQAEPEKEDIMVMDTKLKIIEILQFILNVRLDYRISCLLCIFKREFDESNSQTSE      1019
                                   *********************************************************:**

sp|P11881|ITPR1_MOUSE              TSSGNSSQEGPSNVPGALDFEHIEEQAEGIFGGSEENTPLDLDDHGGRTFLRVLLHLTMH      1070
sp|P29994|ITPR1_RAT                TSSGNSSQEGPSNVPGALDFEHIEEQAEGIFGGSEENTPLDLDDHGGRTFLRVLLHLTMH      1070
sp|Q14643|ITPR1_HUMAN              TSSGNSSQEGPSNVPGALDFEHIEEQAEGIFGGSEENTPLDLDDHGGRTFLRVLLHLTMH      1080
tr|A0A8I3NKB2|A0A8I3NKB2_CANLF     TSSGNSSQEGPSNVPGTLDFEHIEEQAEGIFGGSEENTPLDLDDHGGRTFLRVLLHLTMH      1079
                                   ****************:*******************************************

sp|P11881|ITPR1_MOUSE              DYPPLVSGALQLLFRHFSQRQEVLQAFKQVQLLVTSQDVDNYKQIKQDLDQLRSIVEKSE      1130
sp|P29994|ITPR1_RAT                DYPPLVSGALQLLFRHFSQRQEVLQAFKQVQLLVTSQDVDNYKQIKQDLDQLRSIVEKSE      1130
sp|Q14643|ITPR1_HUMAN              DYPPLVSGALQLLFRHFSQRQEVLQAFKQVQLLVTSQDVDNYKQIKQDLDQLRSIVEKSE      1140
tr|A0A8I3NKB2|A0A8I3NKB2_CANLF     DYPPLVSGALQLLFRHFSQRQEVLQAFKQVQLLVTSQDVDNYKQIKQDLDQLRSIVEKSE      1139
                                   ************************************************************

sp|P11881|ITPR1_MOUSE              LWVYKGQGPDEPMDGASGENEHKKTEEGTSKPLKHESTSSYNYRVVKEILIRLSKLCVQE      1190
sp|P29994|ITPR1_RAT                LWVYKGQGPDEPMDGASGENEHKKTEEGTSKPLKHESTSSYNYRVVKEILIRLSKLCVQE      1190
sp|Q14643|ITPR1_HUMAN              LWVYKGQGPDETMDGASGENEHKKTEEGNNKPQKHESTSSYNYRVVKEILIRLSKLCVQE      1200
tr|A0A8I3NKB2|A0A8I3NKB2_CANLF     LWVYKGQGPDEAMDGASGENEHKKTEEGHNKSQKHESTSSYNYRVVKEILIRLSKLCVQE      1199
                                   *********** **************** .*  ***************************

sp|P11881|ITPR1_MOUSE              SASVRKSRKQQQRLLRNMGAHAVVLELLQIPYEKAEDTKMQEIMRLAHEFLQNFCAGNQQ      1250
sp|P29994|ITPR1_RAT                SASVRKSRKQQQRLLRNMGAHAVVLELLQIPYEKAEDTKMQEIMRLAHEFLQNFCAGNQQ      1250
sp|Q14643|ITPR1_HUMAN              SASVRKSRKQQQRLLRNMGAHAVVLELLQIPYEKAEDTKMQEIMRLAHEFLQNFCAGNQQ      1260
tr|A0A8I3NKB2|A0A8I3NKB2_CANLF     SASVRKSRKQQQRLLRNMGAHAVVLELLQIPYEKAEDTKMQEIMRLAHEFLQNFCAGNQQ      1259
                                   ************************************************************

sp|P11881|ITPR1_MOUSE              NQALLHKHINLFLNPGILEAVTMQHIFMNNFQLCSEINERVVQHFVHCIETHGRNVQYIK      1310
sp|P29994|ITPR1_RAT                NQALLHKHINLFLNPGILEAVTMQHIFMNNFQLCSEINERVVQHFVHCIETHGRNVQYIK      1310
sp|Q14643|ITPR1_HUMAN              NQALLHKHINLFLNPGILEAVTMQHIFMNNFQLCSEINERVVQHFVHCIETHGRNVQYIK      1320
tr|A0A8I3NKB2|A0A8I3NKB2_CANLF     NQALLHKHINLFLNPGILEAVTMQHIFMNNFQLCSEINERVVQHFVHCIETHGRNVQYIK      1319
                                   ************************************************************

sp|P11881|ITPR1_MOUSE              FLQTIVKAEGKFIKKCQDMVMAELVNSGEDVLVFYNDRASFQTLIQMMRSERDRMDENSP      1370
sp|P29994|ITPR1_RAT                FLQTIVKAEGKFIKKCQDMVMAELVNSGEDVLVFYNDRASFQTLIQMMRSERDRMDENSP      1370
sp|Q14643|ITPR1_HUMAN              FLQTIVKAEGKFIKKCQDMVMAELVNSGEDVLVFYNDRASFQTLIQMMRSERDRMDENSP      1380
tr|A0A8I3NKB2|A0A8I3NKB2_CANLF     FLQTIVKAEGKFIKKCQDMVMAELVNSGEDVLVFYNDRASFQTLIQMMRSERDRMDENSP      1379
                                   ************************************************************

sp|P11881|ITPR1_MOUSE              L-MYHIHLVELLAVCTEGKNVYTEIKCNSLLPLDDIVRVVTHEDCIPEVKIAYINFLNHC      1429
sp|P29994|ITPR1_RAT                LFMYHIHLVELLAVCTEGKNVYTEIKCNSLLPLDDIVRVVTHEDCIPEVKIAYINFLNHC      1430
sp|Q14643|ITPR1_HUMAN              L-MYHIHLVELLAVCTEGKNVYTEIKCNSLLPLDDIVRVVTHEDCIPEVKIAYINFLNHC      1439
tr|A0A8I3NKB2|A0A8I3NKB2_CANLF     L-MYHIHLVELLAVCTEGKNVYTEIKCNSLLPLDDIVRVVTHEDCIPEVKIAYINFLNHC      1438
                                   * **********************************************************
```

```
sp|P11881|ITPR1_MOUSE             YVDTEVEMKEIYTSNHMWKLFENFLVDICRACNNTSDRKHADSILEKYVTEIVMSIVTTF    1489
sp|P29994|ITPR1_RAT               YVDTEVEMKEIYTSNHMWKLFENFLVDICRACNNTSDRKHADSVLEKYVTEIVMSIVTTF    1490
sp|Q14643|ITPR1_HUMAN             YVDTEVEMKEIYTSNHMWKLFENFLVDICRACNNTSDRKHADSILEKYVTEIVMSIVTTF    1499
tr|A0A8I3NKB2|A0A8I3NKB2_CANLF    YVDTEVEMKEIYTSNHMWKLFENFLVDICRACNNTSDRKHADSILEKYVTEIVMSIVTTF    1498
                                  *******************************************:****************

sp|P11881|ITPR1_MOUSE             FSSPFSDQSTTLQTRQPVFVQLLQGVFRVYHCNWLMPSQKASVESCIRVLSDVAKSRAIA    1549
sp|P29994|ITPR1_RAT               FSSPFSDQSTTLQTRQPVFVQLLQGVFRVYHCNWLMPSQKASVESCIRVLSDVAKSRAIA    1550
sp|Q14643|ITPR1_HUMAN             FSSPFSDQSTTLQTRQPVFVQLLQGVFRVYHCNWLMPSQKASVESCIRVLSDVAKSRAIA    1559
tr|A0A8I3NKB2|A0A8I3NKB2_CANLF    FSSPFSDQSTTLQTRQPVFVQLLQGVFRVYHCNWLMPSQKASVESCIRVLSDVAKSRAIA    1558
                                  ************************************************************

sp|P11881|ITPR1_MOUSE             IPVDLDSQVNNLFLKSHNIVQKTALNWRLSARNAARRDSVLAASRDYRNIIERLQDIVSA    1609
sp|P29994|ITPR1_RAT               IPVDLDSQVNNLFLKSHNIVQKTAMNWRLSARNAARRDSVLAASRDYRNIIERLQDIVSA    1610
sp|Q14643|ITPR1_HUMAN             IPVDLDSQVNNLFLKSHSIVQKTAMNWRLSARNAARRDSVLAASRDYRNIIERLQDIVSA    1619
tr|A0A8I3NKB2|A0A8I3NKB2_CANLF    IPVDLDSQVNNLFLKSHNIVQKTAMNWRLTARNAARRDSVLAASRDYRNIIERLQDIVSA    1618
                                  *****************.******:****:******************************

sp|P11881|ITPR1_MOUSE             LEDRLRPLVQAELSVLVDVLHRPELLFPENTDARRKCESGGFICKLIKHTKQLLEENEEK    1669
sp|P29994|ITPR1_RAT               LEDRLRPLVQAELSVLVDVLHRPELLFPENTDARRKCESGGFICKLIKHTKQLLEENEEK    1670
sp|Q14643|ITPR1_HUMAN             LEDRLRPLVQAELSVLVDVLHRPELLFPENTDARRKCESGGFICKLIKHTKQLLEENEEK    1679
tr|A0A8I3NKB2|A0A8I3NKB2_CANLF    LEDRLRPLVQAELSVLVDVLHRPELLFPENTDARRKCESGGFICKLIKHTKQLLEENEEK    1678
                                  ************************************************************

sp|P11881|ITPR1_MOUSE             LCIKVLQTLREMMTKDRGYGEKQISIDESENAELPQAPEAENSTEQELEPSPPLRQLEDH    1729
sp|P29994|ITPR1_RAT               LCIKVLQTLREMMTKDRGYGEKQISIDELENAELPQPPEAENSTEQELEPSPPLRQLEDH    1730
sp|Q14643|ITPR1_HUMAN             LCIKVLQTLREMMTKDRGYGEKLISIDELDNAELPPAPDSENAT-EELEPSPPLRQLEDH    1738
tr|A0A8I3NKB2|A0A8I3NKB2_CANLF    LCIKVLQTLREMMTKDRGYGEKLISIDELDNAELPQAPDSENST-EELEPSPPLRQLEDH    1737
                                  ********************** ***** :*****  *::**:* :**************

sp|P11881|ITPR1_MOUSE             KRGEALRQILVNRYYGNIRPSGRRESLTSFGNGPLSPGGPSKPGGGGGGPGSSSTSRGEM    1789
sp|P29994|ITPR1_RAT               KRGEALRQILVNRYYGNIRPSGRRESLTSFGNGPLSPGGPSKPGGGGGGPGSGSTSRGEM    1790
sp|Q14643|ITPR1_HUMAN             KRGEALRQVLVNRYYGNVRPSGRRESLTSFGNGPLSAGGPGKPGGGGGGSGSSSMSRGEM    1798
tr|A0A8I3NKB2|A0A8I3NKB2_CANLF    KRGEALRQILVNRYYGNIRPSGRRESLTSFGNGPLSPGGPSKPGGGGGGSGSSSTSRGEM    1797
                                  ********:********:****************** ***.******** **.* *****

sp|P11881|ITPR1_MOUSE             SLAEVQCHLDKEGASNLVIDLIMNASSDRVFHESILLAIALLEGGNTTIQHSFFCRLTED    1849
sp|P29994|ITPR1_RAT               SLAEVQCHLDKEGASNLVIDLIMNASSDRVFHESILLAIALLEGGNTTIQHSFFCRLTED    1850
sp|Q14643|ITPR1_HUMAN             SLAEVQCHLDKEGASNLVIDLIMNASSDRVFHESILLAIALLEGGNTTIQHSFFCRLTED    1858
tr|A0A8I3NKB2|A0A8I3NKB2_CANLF    SLAEVQCHLDKEGASNLVIDLIMNASSDRVFHESILLAIALLEGGNTTIQHSFFCRLTED    1857
                                  ************************************************************

sp|P11881|ITPR1_MOUSE             KKSEKFFKVFYDRMKVAQQEIKATVTVNTSDLGNKKKDDEVDRDAPSRKKAKEPTTQITE    1909
sp|P29994|ITPR1_RAT               KKSEKFFKVFYDRMKVAQQEIKATVTVNTSDLGNKKKDDEVDRDAPSRKKAKEPTTQITE    1910
sp|Q14643|ITPR1_HUMAN             KKSEKFFKVFYDRMKVAQQEIKATVTVNTSDLGNKKKDDEVDRDAPSRKKAKEPTTQITE    1918
tr|A0A8I3NKB2|A0A8I3NKB2_CANLF    KKSEKFFKVFYDRMKVAQQEIKATVTVNTSDLGNKKKDDETDRDAPSRKKAKEPSTQITE    1917
                                  ****************************************.*************:*****

sp|P11881|ITPR1_MOUSE             EVRDQLLEASAATRKAFTTFRREADPDDHYQSGEGTQATTDKAKDDLEMSAVITIMQPIL    1969
sp|P29994|ITPR1_RAT               EVRDQLLEASAATRKAFTTFRREADPDDHYQSGEGTQATTDKAKDDLEMSAVITIMQPIL    1970
sp|Q14643|ITPR1_HUMAN             EVRDQLLEASAATRKAFTTFRREADPDDHYQPGEGTQATADKAKDDLEMSAVITIMQPIL    1978
tr|A0A8I3NKB2|A0A8I3NKB2_CANLF    EARDQLLEASAATRKAFSTFRREADPDDHYQSGEGAQATADKTKDELEMSAVITIMQPIL    1977
                                  *.***************:************* ***:***:**:**:**************

sp|P11881|ITPR1_MOUSE             RFLQLLCENHNRDLQNFLRCQNNKTNYNLVCETLQFLDCICGSTTGGLGLLGLYINEKNV    2029
sp|P29994|ITPR1_RAT               RFLQLLCENHNRDLQNFLRCQNNKTNYNLVCETLQFLDCICGSTTGGLGLLGLYINEKNV    2030
sp|Q14643|ITPR1_HUMAN             RFLQLLCENHNRDLQNFLRCQNNKTNYNLVCETLQFLDCICGSTTGGLGLLGLYINEKNV    2038
tr|A0A8I3NKB2|A0A8I3NKB2_CANLF    RFLQLLCENHNRDLQNFLRCQNNKTNYNLVCETLQFLDCICGSTTGGLGLLGLYINEKNV    2037
                                  ************************************************************

sp|P11881|ITPR1_MOUSE             ALINQTLESLTEYCQGPCHENQNCIATHESNGIDIITALILNDINPLGKKRMDLVLELKN    2089
sp|P29994|ITPR1_RAT               ALINQTLESLTEYCQGPCHENQNCIATHESNGIDIITALILNDINPLGKKRMDLVLELKN    2090
sp|Q14643|ITPR1_HUMAN             ALINQTLESLTEYCQGPCHENQNCIATHESNGIDIITALILNDINPLGKKRMDLVLELKN    2098
tr|A0A8I3NKB2|A0A8I3NKB2_CANLF    ALINQTLESLTEYCQGPCHENQNCIATHESNGIDIITALILNDINPLGKKRMDLVLELKN    2097
                                  ************************************************************

sp|P11881|ITPR1_MOUSE             NASKLLLAIMESRHDSENAERILYNMRPKELVEVIKKAYMQGEVEFEDGENGEDGAASPR    2149
sp|P29994|ITPR1_RAT               NASKLLLAIMESRHDSENAERILYNMRPKELVEVIKKAYMQGEVEFEDGENGEDGAASPR    2150
sp|Q14643|ITPR1_HUMAN             NASKLLLAIMESRHDSENAERILYNMRPKELVEVIKKAYMQGEVEFEDGENGEDGAASPR    2158
tr|A0A8I3NKB2|A0A8I3NKB2_CANLF    NASKLLLAIMESRHDSENAERILYNMRPKELVEVIKKAYMQGEVEFEDGENGEDGAASPR    2157
                                  ************************************************************

sp|P11881|ITPR1_MOUSE             NVGHNIYILAHQLARHNKELQTMLKPGGQVDGDEALEFYAKHTAQIEIVRLDRTMEQIVF    2209
sp|P29994|ITPR1_RAT               NVGHNIYILAHQLARHNKELQTMLKPGGQVDGDEALEFYAKHTAQIEIVRLDRTMEQIVF    2210
sp|Q14643|ITPR1_HUMAN             NVGHNIYILAHQLARHNKELQSMLKPGGQVDGDEALEFYAKHTAQIEIVRLDRTMEQIVF    2218
tr|A0A8I3NKB2|A0A8I3NKB2_CANLF    NVGHNIYILAHQLARHNKELQTMLKPGGQVDGDEALEFYAKHTAQIEIVRLDRTMEQIVF    2217
                                  *********************:**************************************

sp|P11881|ITPR1_MOUSE             PVPSICEFLTKESKLRIYYTTERDEQGSKINDFFLRSEDLFNEMNWQKKLRAQPVLYWCA    2269
sp|P29994|ITPR1_RAT               PVPSICEFLTKESKLRIYYTTERDEQGSKINDFFLRSEDLFNEMNWQKKLRAQPVLYWCA    2270
sp|Q14643|ITPR1_HUMAN             PVPSICEFLTKESKLRIYYTTERDEQGSKINDFFLRSEDLFNEMNWQKKLRAQPVLYWCA    2278
tr|A0A8I3NKB2|A0A8I3NKB2_CANLF    PVPSICEFLTKESKLRIYYTTERDEQGSKINDFFLRSEDLFNEMNWQKKLRAQPVLYWCA    2277
                                  ************************************************************

sp|P11881|ITPR1_MOUSE             RNMSFWSSISFNLAVLMNLLVAFFYPFKGVRGGTLEPHWSGLLWTAMLISLAIVIALPKP    2329
sp|P29994|ITPR1_RAT               RNMSFWSSISFNLAVLMNLLVAFFYPFKGVRGGTLEPHWSGLLWTAMLISLAIVIALPKP    2330
sp|Q14643|ITPR1_HUMAN             RNMSFWSSISFNLAVLMNLLVAFFYPFKGVRGGTLEPHWSGLLWTAMLISLAIVIALPKP    2338
tr|A0A8I3NKB2|A0A8I3NKB2_CANLF    RNMSFWSSISFNLAVLMNLLVAFFYPFKGVRGGTLEPHWSGLLWTAMLISLAIVIALPKP    2337
                                  ************************************************************

sp|P11881|ITPR1_MOUSE             HGIRALIASTILRLIFSVGLQPTLFLLGAFNVCNKIIFLMSFVGNCGTFTRGYRAMVLDV    2389
sp|P29994|ITPR1_RAT               HGIRALIASTILRLIFSVGLQPTLFLLGAFNVCNKIIFLMSFVGNCGTFTRGYRAMVLDV    2390
sp|Q14643|ITPR1_HUMAN             HGIRALIASTILRLIFSVGLQPTLFLLGAFNVCNKIIFLMSFVGNCGTFTRGYRAMVLDV    2398
tr|A0A8I3NKB2|A0A8I3NKB2_CANLF    HGIRALIASTILRLIFSVGLQPTLFLLGAFNVCNKIIFLMSFVGNCGTFTRGYRAMVLDV    2397
                                  ************************************************************

sp|P11881|ITPR1_MOUSE             EFLYHLLYLLICAMGLFVHEFFYSLLLFDLVYREETLLNVIKSVTRNGRSIILTAVLALI    2449
sp|P29994|ITPR1_RAT               EFLYHLLYLLICAMGLFVHEFFYSLLLFDLVYREETLLNVIKSVTRNGRPIILTAALALI    2450
sp|Q14643|ITPR1_HUMAN             EFLYHLLYLVICAMGLFVHEFFYSLLLFDLVYREETLLNVIKSVTRNGRSIILTAVLALI    2458
tr|A0A8I3NKB2|A0A8I3NKB2_CANLF    EFLYHLLYLLICAMGLFVHEFFYSLLLFDLVYREETLLNVIKSVTRNGRSIILTAVLALI    2457
                                  *********:*************************************** *****.****
```

```
sp|P11881|ITPR1_MOUSE               LVYLFSIVGYLFFKDDFILEVDRLPNETAVPETGESLANDFLYSDVCRVETGENCTSPAP         2509
sp|P29994|ITPR1_RAT                 LVYLFSIVGYLFFKDDFILEVDRLPNETAGPETGESLANDFLYSDVCRVETGENCTSPAP         2510
sp|Q14643|ITPR1_HUMAN               LVYLFSIVGYLFFKDDFILEVDRLPNETAVPETGESLASEFLFSDVCRVESGENCSSPAP         2518
tr|A0A8I3NKB2|A0A8I3NKB2_CANLF      LVYLFSIVGYLFFKDDFILEVDRLPNETALPEAGESLASEFLYSDVCRVETGENCSSPAP         2517
                                    ***************************** **:*****.:**:*******:****:****

sp|P11881|ITPR1_MOUSE               KEELLPAEETEQDKEHTCETLLMCIVTVLSHGLRSGGGVGDVLRKPSKEEPLFAARVIYD         2569
sp|P29994|ITPR1_RAT                 KEELLPVEETEQDKEHTCETLLMCIVTVLSHGLRSGGGVGDVLRKPSKEEPLFAARVIYD         2570
sp|Q14643|ITPR1_HUMAN               REELVPAEETEQDKEHTCETLLMCIVTVLSHGLRSGGGVGDVLRKPSKEEPLFAARVIYD         2578
tr|A0A8I3NKB2|A0A8I3NKB2_CANLF      KEELVLAEETEQDKEHTCETLLMCIVTVLSHGLRSGGGVGDVLRKPSKEEPLFAARVIYD         2577
                                    :***: .*****************************************************

sp|P11881|ITPR1_MOUSE               LLFFFMVIIIVLNLIFGVIIDTFADLRSEKQKKEEILKTTCFICGLERDKFDNKTVTFEE         2629
sp|P29994|ITPR1_RAT                 LLFFFMVIIIVLNLIFGVIIDTFADLRSEKQKKEEILKTTCFICGLERDKFDNKTVTFEE         2630
sp|Q14643|ITPR1_HUMAN               LLFFFMVIIIVLNLIFGVIIDTFADLRSEKQKKEEILKTTCFICGLERDKFDNKTVTFEE         2638
tr|A0A8I3NKB2|A0A8I3NKB2_CANLF      LLFFFMVIIIVLNLIFGVIIDTFADLRSEKQKKEEILKTTCFICGLERDKFDNKTVTFEE         2637
                                    ************************************************************

sp|P11881|ITPR1_MOUSE               HIKEEHNMWHYLCFIVLVKVKDSTEYTGPESYVAEMIRERNLDWFPRMRAMSLVSSDSEG         2689
sp|P29994|ITPR1_RAT                 HIKEEHNMWHYLCFIVLVKVKDSTEYTGPESYVAEMIRERNLDWFPRMRAMSLVSSDSEG         2690
sp|Q14643|ITPR1_HUMAN               HIKEEHNMWHYLCFIVLVKVKDSTEYTGPESYVAEMIKERNLDWFPRMRAMSLVSSDSEG         2698
tr|A0A8I3NKB2|A0A8I3NKB2_CANLF      HIKEEHNMWHYLCFIVLVKVKDSTEYTGPESYVAEMIKERNLDWFPRMRAMSLVSSDSEG         2697
                                    *************************************:**********************

sp|P11881|ITPR1_MOUSE               EQNELRNLQEKLESTMKLVTNLSGQLSELKDQMTEQRKQKQRIGLLGHPPHMNVNPQQPA         2749
sp|P29994|ITPR1_RAT                 EQNELRNLQEKLESTMKLVTNLSGQLSELKDQMTEQRKQKQRIGLLGHPPHMNVNPQQPA         2750
sp|Q14643|ITPR1_HUMAN               EQNELRNLQEKLESTMKLVTNLSGQLSELKDQMTEQRKQKQRIGLLGHPPHMNVNPQQPA         2758
tr|A0A8I3NKB2|A0A8I3NKB2_CANLF      EQNELRNLQEKLESTMKLVTNLSGQLSELKDQMTEQRKQKQRIGLLGHPPHMNVNPQQPA         2757
                                    ************************************************************
```

Alignment of the dog ITPR2 protein sequence (A0A8I3PW88) to human (Q14571), mouse (Q9Z329) and rat (P29995) ITPR2 with the Clustal Omega algorithm (CLUSTAL O(1.2.4)).

```
sp|Q9Z329|ITPR2_MOUSE               MSDKMSSFLYIGDIVSLYAEGSVNGFISTLGLVDDRCVVHPEAGDLANPPKKFRDCLFKV         60
sp|P29995|ITPR2_RAT                 MSDKMSSFLYIGDIVSLYAEGSVNGFISTLGLVDDRCVVHPEAGDLTNPPKKFRDCLFKV         60
tr|A0A8I3PW88|A0A8I3PW88_CANLF      MSDKMSSFLYIGDIVSLYAEGSVNGFISTLGLVDDRCVVHPEAGDLANPPKKFRDCLFKV         60
sp|Q14571|ITPR2_HUMAN               MTEKMSSFLYIGDIVSLYAEGSVNGFISTLGLVDDRCVVHPEAGDLANPPKKFRDCLFKV         60
                                    *::*******************************************:*************

sp|Q9Z329|ITPR2_MOUSE               CPMNRYSAQKQYWKAKQAKQGNHTEAALLKKLQHAAELEQKQNESENRKLLGEIVKYSNV         120
sp|P29995|ITPR2_RAT                 CPMNRYSAQKQYWKAKQAKQGNHTEAALLKKLQHAAELEQKQNESENRKLLGEIVKYSKV         120
tr|A0A8I3PW88|A0A8I3PW88_CANLF      CPMNRYSAQKQYWKAKQAKQGNHTEAALLKKLQHAAELEQKQNESENKKLLGEIVKYSNV         120
sp|Q14571|ITPR2_HUMAN               CPMNRYSAQKQYWKAKQAKQGNHTEAALLKKLQHAAELEQKQNESENKKLLGEIVKYSNV         120
                                    ***********************************************:**********:*

sp|Q9Z329|ITPR2_MOUSE               IQLLHIKSNKYLTVNKRLPALLEKNAMRVSLDAAGNEGSWFYIHPFWKLRSEGDNIVVGD         180
sp|P29995|ITPR2_RAT                 IQLLHIKSNKYLTVNKRLPALLEKNAMRVSLDAAGNEGSWFYIHPFWKLRSEGDNIVVGD         180
tr|A0A8I3PW88|A0A8I3PW88_CANLF      IQLLHIKSNKYLTVNKRLPALLEKNAMRVSLDAAGNEGSWFYIHPFWKLRSEGDNIVVGD         180
sp|Q14571|ITPR2_HUMAN               IQLLHIKSNKYLTVNKRLPALLEKNAMRVSLDAAGNEGSWFYIHPFWKLRSEGDNIVVGD         180
                                    ************************************************************

sp|Q9Z329|ITPR2_MOUSE               KVVLMPVNAGQPLHASNVELLDNPGCKEVNAVNCNTSWKITLFMKFSSYREDVLKGGDVV         240
sp|P29995|ITPR2_RAT                 KVVLMPVNAGQPLHASNVELLDNPGCKEVNAVNCNTSWKITLFMKFSSYREDVLKGGDVV         240
tr|A0A8I3PW88|A0A8I3PW88_CANLF      KVVLMPVNAGQPLHASNIELLDNPGCKEVNAVNCNTSWKITLFMKYSSYREDVLKGGDVV         240
sp|Q14571|ITPR2_HUMAN               KVVLMPVNAGQPLHASNIELLDNPGCKEVNAVNCNTSWKITLFMKYSSYREDVLKGGDVV         240
                                    *****************:***************************:**************

sp|Q9Z329|ITPR2_MOUSE               RLFHAEQEKFLTCDDYEKKQHIFLRTTLRQSATSATSSKALWEIEVVHHDPCRGGAGQWN         300
sp|P29995|ITPR2_RAT                 RLFHAEQEKFLTCDDYEKKQHIFLRTTLRQSATSATSSKALWEIEVVHHDPCRGGAGQWN         300
tr|A0A8I3PW88|A0A8I3PW88_CANLF      RLFHAEQEKFLTCDEYEKKQHIFLRTTLRQSATSATSSKALWEIEVVHHDPCRGGAGQWN         300
sp|Q14571|ITPR2_HUMAN               RLFHAEQEKFLTCDEYEKKQHIFLRTTLRQSATSATSSKALWEIEVVHHDPCRGGAGQWN         300
                                    **************:*********************************************

sp|Q9Z329|ITPR2_MOUSE               SLFRFKHLATGNYLAAELNPDYRDAQNEGKNVKDGEIPTPKKKRQAGEKIMYTLVSVPHG         360
sp|P29995|ITPR2_RAT                 SLFRFKHLATGNYLAAELNPDYRDAQNEGKTVRDGELPTSKKKHQAGEKIMYTLVSVPHG         360
tr|A0A8I3PW88|A0A8I3PW88_CANLF      SLFRFKHLATGNYLAAELNPDYRDAQNEGKNLRDGDLPTSKKKRQAGEKIMYTLVSVPHG         360
sp|Q14571|ITPR2_HUMAN               SLFRFKHLATGNYLAAELNPDYRDAQNEGKNVRDGVPPTSKKKRQAGEKIMYTLVSVPHG         360
                                    ******************************.::**  ** ***:****************

sp|Q9Z329|ITPR2_MOUSE               NDIASLFELDATTLQRADCLVPRNSYVRLRHLCTNTWVTSTTIPIDTEEERPVMLKIGTC         420
sp|P29995|ITPR2_RAT                 NDIASLFELDATTLQRADCLVPRNSYVRLRHLCTNTWVTSTSIPIDTEEERPVMLKIGTC         420
tr|A0A8I3PW88|A0A8I3PW88_CANLF      NDIASLFELDATTLQRADCLVPRNSYVRLRHLCTNTWVTSTSIPIDTDEERPVMLKIGTC         420
sp|Q14571|ITPR2_HUMAN               NDIASLFELDATTLQRADCLVPRNSYVRLRHLCTNTWVTSTSIPIDTDEERPVMLKIGTC         420
                                    *****************************************:*****:************

sp|Q9Z329|ITPR2_MOUSE               QTKEDKEAFAIVCVPLSEVRDLDFANDANKVLATTVKKLENGSITQNERRFVTKLLEDLI         480
sp|P29995|ITPR2_RAT                 QTKEDKEAFAIVCVPLSEVRDLDFANDANKVLATTVKKLENGSITQNERRFVTKLLEDLI         480
tr|A0A8I3PW88|A0A8I3PW88_CANLF      QTKEDKEAFAIVSVPLSEVRDLDFANDANKVLATTVKKLENGTITQNERRFVTKLLEDLI         480
sp|Q14571|ITPR2_HUMAN               QTKEDKEAFAIVSVPLSEVRDLDFANDANKVLATTVKKLENGTITQNERRFVTKLLEDLI         480
                                    ************.*****************************:*****************

sp|Q9Z329|ITPR2_MOUSE               FFVADVTNNGQDVLDVVITKPNRERQKLMREQNILAQVFGILKAPFKEKAGEGSMLRLED         540
sp|P29995|ITPR2_RAT                 FFVADVTNNGQDVLDVVITKPNRERQKLMREQNILAQVFGILKAPFKEKAGEGSMLRLED         540
tr|A0A8I3PW88|A0A8I3PW88_CANLF      FFVADVLNNGQEVLDVVITKPNRERQKLMREQNILAQVFGILKAPFKEKAGEGSMLRLED         540
sp|Q14571|ITPR2_HUMAN               FFVADVPNNGQEVLDVVITKPNRERQKLMREQNILAQVFGILKAPFKEKAGEGSMLRLED         540
                                    ****** ****:************************************************

sp|Q9Z329|ITPR2_MOUSE               LGDQRYAPYKYVLRLCYRVLRHSQQDYRKNQEYIAKNFCVMQSQIGYDILAEDTITALLH         600
sp|P29995|ITPR2_RAT                 LGDQRYAPYKYVLRLCYRVLRHSQQDYRKNQEYIAKNFCVMQSQIGYDILAEDTITALLH         600
tr|A0A8I3PW88|A0A8I3PW88_CANLF      LGDQRYAPYKYMLRLCYRVLRHSQQDYRKNQEYIAKNFCVMQSQIGYDILAEDTITALLH         600
sp|Q14571|ITPR2_HUMAN               LGDQRYAPYKYMLRLCYRVLRHSQQDYRKNQEYIAKNFCVMQSQIGYDILAEDTITALLH         600
                                    ***********:************************************************

sp|Q9Z329|ITPR2_MOUSE               NNRKLLEKHITAKEIETFVSLLRRNREPRFLDYLSDLCVSNSTAIPVTQELICKFMLSPG         660
sp|P29995|ITPR2_RAT                 NNRKLLEKHITAKEIETFVSLLRRNREPRFLDYLSDLCVSNSTAIPVTQELICKFMLSPG         660
tr|A0A8I3PW88|A0A8I3PW88_CANLF      NNRKLLEKHITAKEIETFVSLLRRNREPRFLDYLSDLCVSNTTAIPVTQELICKFMLSPG         660
sp|Q14571|ITPR2_HUMAN               NNRKLLEKHITAKEIETFVSLLRRNREPRFLDYLSDLCVSNTTAIPVTQELICKFMLSPG         660
                                    *****************************************:******************
```

```
sp|Q9Z329|ITPR2_MOUSE                 NADILIQTKLVSMQVENPMESSILPDDIDDEEVWLYWIDSNKEPHGKAIRHLAQEAREGT      720
sp|P29995|ITPR2_RAT                   NADILIQTKLVSMQVENPMESSILPDDIDDEEVWLYWIDSNKEPHGKAIRHLAQEAREGT      720
tr|A0A8I3PW88|A0A8I3PW88_CANLF        NADILIQTKLVSMQVDNPMESSILSDDIDDEEVWLYWIDSNKEPHGKAIRHLAQEAKEGT      720
sp|Q14571|ITPR2_HUMAN                 NADILIQTKVVSMQADNPMESSILSDDIDDEEVWLYWIDSNKEPHGKAIRHLAQEAKEGT      720
                                      *********:****.:******** *******************************:***

sp|Q9Z329|ITPR2_MOUSE                 KADLEVLTYYRYQLNLFARMCLDRQYLAINQISTQLSVDLILRCVSDESLPFDLRASFCR      780
sp|P29995|ITPR2_RAT                   KADLEVLTYYRYQLNLFARMCLDRQYLAINQISTQLSVDLILRCVSDESLPFDLRASFCR      780
tr|A0A8I3PW88|A0A8I3PW88_CANLF        KADLEVLTYYRYQLNLFARMCLDRQYLAINQISTQLSVDLILRCVSDESLPFDLRASFCR      780
sp|Q14571|ITPR2_HUMAN                 KADLEVLTYYRYQLNLFARMCLDRQYLAINQISTQLSVDLILRCVSDESLPFDLRASFCR      780
                                      ************************************************************

sp|Q9Z329|ITPR2_MOUSE                 LMLHMHVDRDPQESVVPVRYARLWTEIPTKITIHEYDSITDSSRNDMKRKFALTMEFVEE      840
sp|P29995|ITPR2_RAT                   LMLHMHVDRDPQESVVPVRYARLWTEIPTKITIHEYDSITDSSRNDMKRKFALTMEFVEE      840
tr|A0A8I3PW88|A0A8I3PW88_CANLF        LMLHMHVDRDPQESVVPVRYARLWTEIPTKITIHEYDSITDSSRNDMKRKFALTMEFVEE      840
sp|Q14571|ITPR2_HUMAN                 LMLHMHVDRDPQESVVPVRYARLWTEIPTKITIHEYDSITDSSRNDMKRKFALTMEFVEE      840
                                      ************************************************************

sp|Q9Z329|ITPR2_MOUSE                 YLKEVVNQPFPFGDKEKNKLTFEVVHLARNLIYFGFYSFSELLRLTRTLLAILDIVQAPM      900
sp|P29995|ITPR2_RAT                   YLKEVVNQPFPFGDKEKNKLTFEVVHLARNLIYFGFYSFSELLRLTRTLLAILDIVQAPM      900
tr|A0A8I3PW88|A0A8I3PW88_CANLF        YLKEVVNQPFPFGDKEKNKLTFEVVHLARNLIYFGFYSFSELLRLTRTLLAILDIVQAPM      900
sp|Q14571|ITPR2_HUMAN                 YLKEVVNQPFPFGDKEKNKLTFEVVHLARNLIYFGFYSFSELLRLTRTLLAILDIVQAPM      900
                                      ************************************************************

sp|Q9Z329|ITPR2_MOUSE                 SSYFERLSKFQDGSNNVMRTIHGVGEMMTQMVLSRGSIFPVSVPDAQPIVHPSKQASPGE      960
sp|P29995|ITPR2_RAT                   SSYFERLSKFQDGSNNVMRTIHGVGEMMTQMVLSRGSIFPVSWPDAQPSVHPSKQASPGE      960
tr|A0A8I3PW88|A0A8I3PW88_CANLF        SSYFERLSKFQDGGNNVMRTIHGVGEMMTQMVLSRGSIFPMSVPDIQPSIHPSKQGSPVE      960
sp|Q14571|ITPR2_HUMAN                 SSYFERLSKFQDGGNNVMRTIHGVGEMMTQMVLSRGSIFPMSVPDVPPSIHPSKQGSPTE      960
                                      *************.**************************:* **  * :*****.** *

sp|Q9Z329|ITPR2_MOUSE                 QEDVTVMDTKLKVIEILQFILSVRLDYRISYMLSIYKKEFGDNNDNGDPSASGTPDTLLP      1020
sp|P29995|ITPR2_RAT                   QEDVTVMDTKLKVIEILQFILSVRLDYRISYMLSIYKKEFGENDGNGDPSASGTPETLLP      1020
tr|A0A8I3PW88|A0A8I3PW88_CANLF        HEDVTVMDTKLKIIEILQFILSVRLDYRISYMLSIYKKEFGENNENTETSANGSPDTLLP      1020
sp|Q14571|ITPR2_HUMAN                 HEDVTVMDTKLKIIEILQFILSVRLDYRISYMLSIYKKEFGEDNDNAETSASGSPDTLLP      1020
                                      :***********:****************************::: * : **.*:*:****

sp|Q9Z329|ITPR2_MOUSE                 SALVPDIDEIAAQAETMFAGRKEKTPVQLDDEGGRTFLRVLIHLIMHDYAPLLSGALQLL      1080
sp|P29995|ITPR2_RAT                   SALVPDIDEIAAQAETMFAGRKEKTPVQLDDEGGRTFLRVLIHLIMHDYAPLLSGALQLL      1080
tr|A0A8I3PW88|A0A8I3PW88_CANLF        SAIVPDIDEIAAQAETMFAGRKEKNPVQLDDEGGRTFLRVLIHLIMHDYPPLLSGALQLL      1080
sp|Q14571|ITPR2_HUMAN                 SAIVPDIDEIAAQAETMFAGRKEKNPVQLDDEGGRTFLRVLIHLIMHDYPPLLSGALQLL      1080
                                      **:*********************.************************ **********

sp|Q9Z329|ITPR2_MOUSE                 FKHFSQRAEVLQAFKQVQLLVSNQDVDNYKQIKADLDQLRLTVEKSELWVEKSGSYENGD      1140
sp|P29995|ITPR2_RAT                   FKHFSQRAEVLQAFKQVQLLVSNQDVDNYKQIKADLDQLRLTVEKSELWVEKSGSYENGD      1140
tr|A0A8I3PW88|A0A8I3PW88_CANLF        FKHFSQRAEVLQAFKQVQLLVSNQDVDNYKQIKADLDQLRLTVEKSELWVEKSSSYENGE      1140
sp|Q14571|ITPR2_HUMAN                 FKHFSQRAEVLQAFKQVQLLVSNQDVDNYKQIKADLDQLRLTVEKSELWVEKSSNYENGE      1140
                                      *****************************************************..****:

sp|Q9Z329|ITPR2_MOUSE                 VGEGQAKGGEEANEESNLLSPVQDGAKTPQIDSNKGNNYRIVKEILIRLSKLCVQNKKCR      1200
sp|P29995|ITPR2_RAT                   MGEGQAKGGEEANEESNLLSPVQDGAKTPQIDSNKGNNYRIVKEILIRLSKLCVQNKKCR      1200
tr|A0A8I3PW88|A0A8I3PW88_CANLF        MGESQVKGGEEPIEESSILSPVQDGTRKPQIDSNKSNNYRIVKEILIRLSKLCVQNKKCR      1200
sp|Q14571|ITPR2_HUMAN                 IGESQVKGGEEPIEESNILSPVQDGTKKPQIDSNKSNNYRIVKEILIRLSKLCVQNKKCR      1200
                                      :**.*.*****  ***.:*******::.*******.************************

sp|Q9Z329|ITPR2_MOUSE                 NQHQRLLKNMGAHSVVLDLLQIPYEKTDEKMNEVMDLAHTFLQNFCRGNPQNQVLLHKHL      1260
sp|P29995|ITPR2_RAT                   NQHQRLLKNMGAHSVVLDLLQIPYEKTDEKMNEVMDLAHTFLQNFCRGNPQNQVLLHKHL      1260
tr|A0A8I3PW88|A0A8I3PW88_CANLF        NQHQRLLKNMGAHSVVLDLLKIPYEKNDEKMNEVMNLAHTFLQNFCRGNPQNQVLLHKHL      1260
sp|Q14571|ITPR2_HUMAN                 NQHQRLLKNMGAHSVVLDLLQIPYEKNDEKMNEVMNLAHTFLQNFCRGNPQNQVLLHKHL      1260
                                      ********************:*****.********:************************

sp|Q9Z329|ITPR2_MOUSE                 NLFLTPGLLEAETMRHIFMNNYHLCNEISERVVQHFVHCIETHGRHVEYLRFLQTIVKAD      1320
sp|P29995|ITPR2_RAT                   NLFLTPGLLEAETMRHIFMNNYHLCNEISERVVQHFVHCIETHGRHVEYLRFLQTIVKAD      1320
tr|A0A8I3PW88|A0A8I3PW88_CANLF        NLFLTPGLLEAETMRHIFMNNYHLCNEISERVVQHFVHCIETHGRHVEYLRFLQTIVKAD      1320
sp|Q14571|ITPR2_HUMAN                 NLFLTPGLLEAETMRHIFMNNYHLCNEISERVVQHFVHCIETHGRHVEYLRFLQTIVKAD      1320
                                      ************************************************************

sp|Q9Z329|ITPR2_MOUSE                 GKYVKKCQDMVMTELINGGEDVLIFYNDRASFPILLNMMCSERARGDESGPLAYHITLVE      1380
sp|P29995|ITPR2_RAT                   GKYVKKCQDMVMTELINGGEDVLIFYNDRASFPILLNMMCSERARGDESGPLAYHITLVE      1380
tr|A0A8I3PW88|A0A8I3PW88_CANLF        GKYVKKCQDMVMTELINGGEDVLIFYNDRASFPILLHMMCSERDRGDESGPLAYHITLVE      1380
sp|Q14571|ITPR2_HUMAN                 GKYVKKCQDMVMTELINGGEDVLIFYNDRASFPILLHMMCSERDRGDESGPLAYHITLVE      1380
                                      ************************************:****** ****************

sp|Q9Z329|ITPR2_MOUSE                 LLAACTEGKNVYTEIKCNSLLPLDDIVRVVTHDDCIPEVKIAYVNFVNHCYVDTEVEMKE      1440
sp|P29995|ITPR2_RAT                   LLAACTEGKNVYTEIKCNSLLPLDDIVRVVTHDDCIPEVKIAYVNFVNHCYVDTEVEMKE      1440
tr|A0A8I3PW88|A0A8I3PW88_CANLF        LLAACTEGKNVYTEIKCNSLLPLDDIVRVVTHDDCIPEVKIAYVNFVNHCYVDTEVEMKE      1440
sp|Q14571|ITPR2_HUMAN                 LLAACTEGKNVYTEIKCNSLLPLDDIVRVVTHDDCIPEVKIAYVNFVNHCYVDTEVEMKE      1440
                                      ************************************************************

sp|Q9Z329|ITPR2_MOUSE                 IYTSNHIWKLFENFLVDMARVCNTTTDRKHADTFLERCVTESVMNIVSGFFNSPFSDNST      1500
sp|P29995|ITPR2_RAT                   IYTSNHIWKLFENFLVDMARVCNTTTDRKHADTFLERCVTESVMNIVSGFFNSPFSDNST      1500
tr|A0A8I3PW88|A0A8I3PW88_CANLF        IYTSNHIWKLFENFLVDMARVCNTTTDRKHADTFLEKCVTESIMNIVSGFFNSPFSDNST      1500
sp|Q14571|ITPR2_HUMAN                 IYTSNHIWKLFENFLVDMARVCNTTTDRKHADIFLEKCVTESIMNIVSGFFNSPFSDNST      1500
                                      ******************************** ***:*****:*****************

sp|Q9Z329|ITPR2_MOUSE                 SLQTHQPVFIQLLQSAFRIYNCTWPNPAQKASVESCIRALAEVAKNRGIAIPVDLDSQVN      1560
sp|P29995|ITPR2_RAT                   SLQTHQPVFIQLLQSAFRIYNCTWPNPAQKASVESCIRALAEVAKNRGIAIPVDLDSQVN      1560
tr|A0A8I3PW88|A0A8I3PW88_CANLF        SLQTHQPVFIQLLQSAFRIYNCTWPNPAQKSSVESCIRALAEVAKNRGIAIPVDLDSQVN      1560
sp|Q14571|ITPR2_HUMAN                 SLQTHQPVFIQLLQSAFRIYNCTWPNPAQKASVESCIRTLAEVAKNRGIAIPVDLDSQVN      1560
                                      ******************************:*******:*********************

sp|Q9Z329|ITPR2_MOUSE                 TLFMKNHSSTVQRAAMGWRLSARSGPRFKEALGGPAWDYRNIIEKLQDVVASLEQQFSPM      1620
sp|P29995|ITPR2_RAT                   TLFMKNHSSTVQRAAMGWRLSARSGPRFKEALGGPAWDYRNIIEKLQDVVASLEQQFSPM      1620
tr|A0A8I3PW88|A0A8I3PW88_CANLF        TLFLKSHSNMVQRAAMGWRLSARSGPRFKEALGGPAWDYRNIIEKLQDVVASLEQQFSPM      1620
sp|Q14571|ITPR2_HUMAN                 TLFMKSHSNMVQRAAMGWRLSARSGPRFKEALGGPAWDYRNIIEKLQDVVASLEHQFSPM      1620
                                      ***:*.**. ********************************************:*****

sp|Q9Z329|ITPR2_MOUSE                 MQAEFSVLVDVLYSPELLFPEGSDARIRCGAFMSKLINHTKKLMEKEEKLCIKILQTLRE      1680
sp|P29995|ITPR2_RAT                   MQAEFSVLVDVLYSPELLFPEGSDARIRCGAFMSKLINHTKKLMEKEEKLCIKILQTLRE      1680
tr|A0A8I3PW88|A0A8I3PW88_CANLF        MQAEFSVLVDVLYSPELLFPEGSDARIRCGAFMSKLINHTKKLMEKEEKLCIKILQTLRE      1680
sp|Q14571|ITPR2_HUMAN                 MQAEFSVLVDVLYSPELLFPEGSDARIRCGAFMSKLINHTKKLMEKEEKLCIKILQTLRE      1680
                                      ************************************************************
```

```
sp|Q9Z329|ITPR2_MOUSE              MLEKKDSFMEEGSTLRRILLNRYFKGDHSISVNGPLSGAYAKTAQVGGSFSGQDSDKKGI          1740
sp|P29995|ITPR2_RAT                MLEKKDSFMEESSTLRKILLNRYFKGDHSVGVNGPLSGAYAKTAQVGGGFTGQDADKTGI          1740
tr|A0A8I3PW88|A0A8I3PW88_CANLF     MLEKKDSFVEEGNTLRKILLNRYFKGDYGVGMNGHLSGTYCKSAQGGGSFSGQDSDKMGI          1740
sp|Q14571|ITPR2_HUMAN              MLEKKDSFVEEGNTLRKILLNRYFKGDYSIGVNGHLSGAYSKTAQVGGSFSGQDSDKMGI          1740
                                   ********:**..***:**********:.:.:** ***:*.*:** **.*:***:** **

sp|Q9Z329|ITPR2_MOUSE              SMSDIQCLLDKEGASELVIDVIVNTKNDRIFSEGILLGIALLEGGNTQTQYSFYQQLHEQ          1800
sp|P29995|ITPR2_RAT                SMSDIQCLLDKEGASELVIDVIVNTKNDRIFSEGILLGIALLEGGNTQTQNSFYQQLHEQ          1800
tr|A0A8I3PW88|A0A8I3PW88_CANLF     SMSDIQCLLDKEGASELVIDVIVNTKNDRIFSEGILLGIALLEGGNTQTQYSFYQQLHEQ          1800
sp|Q14571|ITPR2_HUMAN              SMSDIQCLLDKEGASELVIDVIVNTKNDRIFSEGIFLGIALLEGGNTQTQYSFYQQLHEQ          1800
                                   ***********************************:************** *********

sp|Q9Z329|ITPR2_MOUSE              KKSEKFFKVLYDRMKAAQKEIRSTVTVNTIDLGSKKREEDSDVMALGPRMRVRDSSLHLR          1860
sp|P29995|ITPR2_RAT                KKSEKFFKVLYDRMKAAQKEIRSTVTVNTIDLGSKKREEDSDLMALGPRMRVRDSSLHLK          1860
tr|A0A8I3PW88|A0A8I3PW88_CANLF     KKSEKFFKVLYDRMKAAQKEIRSTVTVNTIDLGNKKRDDDNELMTAGPRMRVRDSSLLLK          1860
sp|Q14571|ITPR2_HUMAN              KKSEKFFKVLYDRMKAAQKEIRSTVTVNTIDLGNKKRDDDNELMTSGPRMRVRDSTLHLK          1860
                                   *********************************.***::*.::*: *********:* *:

sp|Q9Z329|ITPR2_MOUSE              EGMKGQLTEASSATSKAYCVYRREMDPEIDTMCPGQEAGSAEEKSAEEVTMSPAITIMRP          1920
sp|P29995|ITPR2_RAT                EGMKGQLTEASSATSKAYCVYRREMDPDIDTMCPGQEAGSAEEKSAEEVTMSPAITIMRP          1920
tr|A0A8I3PW88|A0A8I3PW88_CANLF     EGMKGQLTEASSATSKAYCAYRREMDPEIDIMCAGSEAGHAEEKSAEEVTMSPAIAIMQP          1920
sp|Q14571|ITPR2_HUMAN              EGMKGQLTEASSATSKAYCVYRREMDPEIDIMCTGPEAGNTEEKSAEEVTMSPAIAIMQP          1920
                                   *******************.*******:** ** * ***  :***************:**:*

sp|Q9Z329|ITPR2_MOUSE              ILRFLQLLCENHNRELQNFLRNQNNKTNYNLVCETLQFLDCICGSTTGGLGLLGLYINER          1980
sp|P29995|ITPR2_RAT                ILRFLQLLCENHNRELQNFLRNQNNKTNYNLVCETLQFLDCICGSTTGGLGLLGLYINEK          1980
tr|A0A8I3PW88|A0A8I3PW88_CANLF     ILRFLQLLCENHNRELQNFLRNQNNKTNYNLVCETLQFLDCICGSTTGGLGLLGLYINEK          1980
sp|Q14571|ITPR2_HUMAN              ILRFLQLLCENHNRELQNFLRNQNNKTNYNLVCETLQFLDCICGSTTGGLGLLGLYINEK          1980
                                   ***********************************************************:

sp|Q9Z329|ITPR2_MOUSE              NVALVNQTLESLTEYCQGPCHENQTCIATHESNGIDIIIALILNDINPLGKYRMDLVLQL          2040
sp|P29995|ITPR2_RAT                NVALVNQTLESLTEYCQGPCHENQTCIATHESNGIDIIIALILSDINPLGKYRMDLVLQL          2040
tr|A0A8I3PW88|A0A8I3PW88_CANLF     NVVLVNQTLESLTEYCQGPCHENQTCIATHESNGIDIIIALILNDINPLGKYRMDLVLQL          2040
sp|Q14571|ITPR2_HUMAN              NVALVNQNLESLTEYCQGPCHENQTCIATHESNGIDIIIALILNDINPLGKYRMDLVLQL          2040
                                   **.****.***********************************.****************

sp|Q9Z329|ITPR2_MOUSE              KNNASKLLLAIMESRHDSENAERILFNMRPRELVDVMKNAYNQGLECDHGDEEGGDDGVS          2100
sp|P29995|ITPR2_RAT                KNNASKLLLAIMESRHDSENAERILFNMRPKELVDVMKNAYNQGLECNHGDEEGGDDGVS          2100
tr|A0A8I3PW88|A0A8I3PW88_CANLF     KNNASKLLLAIMESRHDSENAERILFNMRPRELVDVMKNAYNQGLECDHGDDEGGDDGVS          2100
sp|Q14571|ITPR2_HUMAN              KNNASKLLLAIMESRHDSENAERILFNMRPRELVDVMKNAYNQGLECDHGDDEGGDDGVS          2100
                                   ******************************:****************:***:********

sp|Q9Z329|ITPR2_MOUSE              PKDVGHNIYILAHQLARHNKLLQQMLKPGSDPEEGDEALKYYANHTAQIEIVRHDRTMEQ          2160
sp|P29995|ITPR2_RAT                PKDVGHNIYILAHQLARHNKLLQQMLKPGSDPEEGDEALKYYANHTAQIEIVRHDRTMEQ          2160
tr|A0A8I3PW88|A0A8I3PW88_CANLF     PKDVGHNIYILAHQLARHNKLLQQMLKPGSDPDDGDEALKYYANHTAQIEIVRHDRTMEQ          2160
sp|Q14571|ITPR2_HUMAN              PKDVGHNIYILAHQLARHNKLLQQMLKPGSDPDEGDEALKYYANHTAQIEIVRHDRTMEQ          2160
                                   ********************************::**************************

sp|Q9Z329|ITPR2_MOUSE              IVFPVPNICEFLTRESKYRVFNTTERDEQGSKVNDFFQQTEDLYNEMKWQKKIRNNPALF          2220
sp|P29995|ITPR2_RAT                IVFPVPNICEFLTRESKYRVFNTTERDEQGSKVNDFFQQTEDLYNEMKWQKKIRNNPALF          2220
tr|A0A8I3PW88|A0A8I3PW88_CANLF     IVFPVPNICEYLTRESKFRVFNTTERDEQGSKVNDFFQQTEDLYNEMKWQKKIRNNPALF          2220
sp|Q14571|ITPR2_HUMAN              IVFPVPNICEYLTRESKCRVFNTTERDEQGSKVNDFFQQTEDLYNEMKWQKKIRNNPALF          2220
                                   **********:****** ******************************************

sp|Q9Z329|ITPR2_MOUSE              WFSRHISLWGSISFNLAVFINLAVALFYPFGDDGDEGTLSPMFSVLLWVAVAICTSMLFF          2280
sp|P29995|ITPR2_RAT                WFSRHISLWGSISFNLAVFINLAVALFYPFGDDGDEGTLSPLFSALLWVAVAICTSMLFF          2280
tr|A0A8I3PW88|A0A8I3PW88_CANLF     WFSRHISLWGSISFNLAVFINLAVALFYPFGDDGDEGTLSPLFSVLLWIAVAICTSMLFF          2280
sp|Q14571|ITPR2_HUMAN              WFSRHISLWGSISFNLAVFINLAVALFYPFGDDGDEGTLSPLFSVLLWIAVAICTSMLFF          2280
                                   *****************************************:**.***:***********

sp|Q9Z329|ITPR2_MOUSE              FSKPVGIRPFLVSVMLRSIYTIGLGPTLILLGAANLCNKIVFLVSFVGNRGTFTRGYRAV          2340
sp|P29995|ITPR2_RAT                FSKPVGIRPFLVSIMLRSIYTIGLGPTLILLGAANLCNKIVFLVSFVGNRGTFTRGYRAV          2340
tr|A0A8I3PW88|A0A8I3PW88_CANLF     FSKPVGIRPFLVSIMLRSIYTIGLGPTLILLGAANLCNKIVFLVSFVGNRGTFTRGYRAV          2340
sp|Q14571|ITPR2_HUMAN              FSKPVGIRPFLVSIMLRSIYTIGLGPTLILLGAANLCNKIVFLVSFVGNRGTFTRGYRAV          2340
                                   *************:**********************************************

sp|Q9Z329|ITPR2_MOUSE              ILDMAFLYHVAYVLVCMLGLFVHEFFYSFLLFDLVYREETLLNVIKSVTRNGRSIILTAV          2400
sp|P29995|ITPR2_RAT                ILDMAFLYHVAYVLVCMLGLFVHEFFYSFLLFDLVYREETLLNVIKSVTRNGRSIILTAV          2400
tr|A0A8I3PW88|A0A8I3PW88_CANLF     ILDMAFLYHVAYVLVCMLGLFVHEFFYSFLLFDLVYREETLLNVIKSVTRNGRSIILTAV          2400
sp|Q14571|ITPR2_HUMAN              ILDMAFLYHVAYVLVCMLGLFVHEFFYSFLLFDLVYREETLLNVIKSVTRNGRSIILTAV          2400
                                   ************************************************************

sp|Q9Z329|ITPR2_MOUSE              LALILVYLFSIIGFLFLKDDFTMEVDRLKNRTPVTGNHGVPTMTLSSMMETCQKENCSPT          2460
sp|P29995|ITPR2_RAT                LALILVYLFSIIGFLFLKDDFTMEVDRLKNRTPVTGNDGVPTMTLTSMLGTCPKENCSPT          2460
tr|A0A8I3PW88|A0A8I3PW88_CANLF     LALILVYLFSIIGFLFLKDDFTMEVDRLKNRTPITGSNEVPTMTLTSMMGTCKNDNCSPT          2460
sp|Q14571|ITPR2_HUMAN              LALILVYLFSIIGFLFLKDDFTMEVDRLKNRTPVTGSHQVPTMTLTTMMEACAKENCSPT          2460
                                   *********************************:**.. ******::*: :* ::*****

sp|Q9Z329|ITPR2_MOUSE              IPSSNTAGEEGEDGIERTCDTLLMCIVTVLNQGLRNGGGVGDVLRRPSKDEPLFAARVVY          2520
sp|P29995|ITPR2_RAT                IPSSNAAGEGGEDGIERTCDTLLMCIVTVLNQGLRNGGGVGDVLRRPSKDEPLFAARVVY          2520
tr|A0A8I3PW88|A0A8I3PW88_CANLF     IPASNTADEEYEDGIERTCDTLLMCIVTVLNQGLRNGGGVGDVLRRPSKDEPLFAARVVY          2520
sp|Q14571|ITPR2_HUMAN              IPASNTADEEYEDGIERTCDTLLMCIVTVLNQGLRNGGGVGDVLRRPSKDEPLFAARVVY          2520
                                   **:**:*.*  *************************************************

sp|Q9Z329|ITPR2_MOUSE              DLLFFFIVIIIVLNLIFGVIIDTFADLRSEKQKKEEILKTTCFICGLERDKFDNKTVSFE          2580
sp|P29995|ITPR2_RAT                DLLFFFIVIIIVLNLIFGVIIDTFADLRSEKQKKEKILKTTCFICGLERDKFDNKTVSFE          2580
tr|A0A8I3PW88|A0A8I3PW88_CANLF     DLLFYFIVIIIVLNLIFGVIIDTFADLRSEKQKKEEILKTTCFICGLERDKFDNKTVSFE          2580
sp|Q14571|ITPR2_HUMAN              DLLFYFIVIIIVLNLIFGVIIDTFADLRSEKQKKEEILKTTCFICGLERDKFDNKTVSFE          2580
                                   ****:******************************:************************

sp|Q9Z329|ITPR2_MOUSE              EHIKSEHNMWHYLYFIVLVKVKDPTEYTGPESYVAQMITEKNLDWFPRMRAMSLVSNEGD          2640
sp|P29995|ITPR2_RAT                EHIKSEHNMWHYLYFIVLVKVKDPTEYTGPESYVAQMITEKNLDWFPRMRAMSLVSNEGD          2640
tr|A0A8I3PW88|A0A8I3PW88_CANLF     EHIKSEHNMWHYLYFLVLVKVKDPTEYTGPESYVAQMIVEKNLDWFPRMRAMSLVSNEGD          2640
sp|Q14571|ITPR2_HUMAN              EHIKSEHNMWHYLYFIVLVKVKDPTEYTGPESYVAQMIVEKNLDWFPRMRAMSLVSNEGD          2640
                                   ***************:**********************.*********************

sp|Q9Z329|ITPR2_MOUSE              SEQNEIRNLQEKLESTMSLVKQLSGQLAELKEQMTEQRKNKQRLGFLGSNTPHVNHHMPP          2700
sp|P29995|ITPR2_RAT                SEQNEIRNLQEKLESTMSLVKQLSGQLAELKEQMTEQRKNKQRLGFLGSNTPHENHHMPP          2700
tr|A0A8I3PW88|A0A8I3PW88_CANLF     SEQNEIRNLQEKLESTMSLVKQLSAQLAELKEQMTEQRKNKQRLGFLGSNTPHVNHHMPP          2700
sp|Q14571|ITPR2_HUMAN              SEQNEIRSLQEKLESTMSLVKQLSGQLAELKEQMTEQRKNKQRLGFLGSNTPHVNHHMPP          2700
                                   *******.****************.**************************** ******
```

```
sp|Q9Z329|ITPR2_MOUSE               H                  2701
sp|P29995|ITPR2_RAT                 H                  2701
tr|A0A8I3PW88|A0A8I3PW88_CANLF      H                  2701
sp|Q14571|ITPR2_HUMAN               H                  2701
                                    *
```

Alignment of the dog ITPR3 protein sequence (XP_038409684.1) to human (NP_002215.2), mouse (P70227) and rat (Q63269) ITPR3 with the Clustal Omega algorithm (CLUSTAL O(1.2.4)).

```
sp|P70227|ITPR3_MOUSE      --------MNEMSSFLHIGDIV---SLYAEGSVNGFISTLGLVDDRCVVEPAAGDLDNPP    49
sp|Q63269|ITPR3_RAT        --------MNEMSSFLHIGDIV---SLYAEGSVNGFISTLGLVDDRCVVEPAAGDLDNPP    49
XP_038409684.1             MRTHRNGGRLSRGAWGHLGLHTSCRWLLCLPATWGAALGTRLVDDRCVVEPAAGDLDNPP    60
NP_002215.2                --------MSEMSSFLHIGDIV---SLYAEGSVNGFISTLGLVDDRCVVEPAAGDLDNPP    49
                                     . .:: *:*  .    * .  :. *      *******************

sp|P70227|ITPR3_MOUSE      KKFRDCLFKVCPMNRYSAQKQYWKAKQTKQDKEKIADVVLLQKLQHAAQMEQKQNDTENK    109
sp|Q63269|ITPR3_RAT        KKFRDCLFKVCPMNRYSAQKQYWKAKQTKQDKEKIADVVLLQKLQHAAQMEQKQNDTENK    109
XP_038409684.1             KKFRDCLFKVCPMNRYSAQKQYWKAKQTKQDKEKIADVVLLQKLQHAAQMEQKQNDTENK    120
NP_002215.2                KKFRDCLFKVCPMNRYSAQKQYWKAKQTKQDKEKIADVVLLQKLQHAAQMEQKQNDTENK    109
                           ************************************************************

sp|P70227|ITPR3_MOUSE      KVHGDVVKYGSVIQLLHMKSNKYLTVNKRLPALLEKNAMRVTLDATGNEGSWLFIQPFWK    169
sp|Q63269|ITPR3_RAT        KVHGDVVKYGSVIQLLHMKSNKYLTVNKRLPALLEKNAMRVTLDATGNEGSWLFIQPFWK    169
XP_038409684.1             KVHGDVVKYGSVIQLLHMKSNKYLTVNKRLPALLEKNAMRVTLDATGNEGSWLFIQPFWK    180
NP_002215.2                KVHGDVVKYGSVIQLLHMKSNKYLTVNKRLPALLEKNAMRVTLDATGNEGSWLFIQPFWK    169
                           ************************************************************

sp|P70227|ITPR3_MOUSE      LRSNGDNVVVGDKVILNPVNAGQPLHASNYELSDNAGCKEVNSVNCNTSWKINLFMQFRD    229
sp|Q63269|ITPR3_RAT        LRSNGDNVVVGDKVILNPVNAGQPLHASNYELSDNVGCKEVNSVNCNTSWKINLFMQFRD    229
XP_038409684.1             LRSNGDNVVVGDKVILNPVNAGQPLHASNYELSDNVGCKEVNSVNCNTSWKINLFMQFRD    240
NP_002215.2                LRSNGDNVVVGDKVILNPVNAGQPLHASNYELSDNAGCKEVNSVNCNTSWKINLFMQFRD    229
                           ***********************************.************************

sp|P70227|ITPR3_MOUSE      HLEEVLKGGDVVRLFHAEQEKFLTCDEYRGKLQVFLRTTLRQSATSATSSNALWEVEVVH    289
sp|Q63269|ITPR3_RAT        HLEEVLKGGDVVRLFHAEQEKFLTCDEYRGKLQVFLRTTLRQSATSATSSNALWEVEVVH    289
XP_038409684.1             HLEEVLKGGDVVRLFHAEQEKFLTCDEYKGKLQVFLRTTLRQSATSATSSNALWEVEVVH    300
NP_002215.2                HLEEVLKGGDVVRLFHAEQEKFLTCDEYKGKLQVFLRTTLRQSATSATSSNALWEVEVVH    289
                           ****************************:*******************************

sp|P70227|ITPR3_MOUSE      HDPCRGGAGHWNGLYRFKHLATGNYLAAEENPSYKGDVSDPKAAGLGAQGRTGRRNAGEK    349
sp|Q63269|ITPR3_RAT        HDPCRGGAGHWNGLYRFKHLATGNYLAAEENPSYKGDVSDPKAAGPGAQSRTGRRNAGEK    349
XP_038409684.1             HDPCRGGAGHWNGLYRFKHLATGNYLAAEENPSYKGDVCEPKAAGMGAQGRIGRRNAGEK    360
NP_002215.2                HDPCRGGAGHWNGLYRFKHLATGNYLAAEENPSYKGDASDPKAAGMGAQGRTGRRNAGEK    349
                           *************************************..:***** ***.* ********

sp|P70227|ITPR3_MOUSE      IKYRLVAVPHGNDIASLFELDPTTLQKTDSFVPRNSYVRLRHLCTNTWIQSTNAPIDVEE    409
sp|Q63269|ITPR3_RAT        IKYRLVAVPHGNDIASLFELDPTTLQKTDSFVPRNSYVRLRHLCTNTWIQSTNAPIDVEE    409
XP_038409684.1             IKYRLVAVPHGNDIASLFELDPTTLQKTDSFVPRNSYVRLRHLCTNTWIQSTNVPIDIEE    420
NP_002215.2                IKYCLVAVPHGNDIASLFELDPTTLQKTDSFVPRNSYVRLRHLCTNTWIQSTNVPIDIEE    409
                           *** *************************************************.***:**

sp|P70227|ITPR3_MOUSE      ERPIRLMLGTCPTKEDKEAFAIVSVPVSEIRDLDFANDASSMLASAVEKLNEGFISQNDR    469
sp|Q63269|ITPR3_RAT        ERPIRLMLGTCPTKEDKEAFAIVSVPVSEIRDLDFANDASSMLASAVEKLNEGFISQNDR    469
XP_038409684.1             ERPIRLMLGTCPTKEDKEAFAIVSVPVSEIRDLDFANDASSMLASAVEKLNEGFISQNDR    480
NP_002215.2                ERPIRLMLGTCPTKEDKEAFAIVSVPVSEIRDLDFANDASSMLASAVEKLNEGFISQNDR    469
                           ************************************************************

sp|P70227|ITPR3_MOUSE      RFVIQLLEDLVFFVSDVPNNGQNVLDIMVTKPNRERQKLMREQNILKQIFGILKAPFRDK    529
sp|Q63269|ITPR3_RAT        RFVIQLLEDLVFFVSDVPNNGQNVLDIMVTKPNRERQKLMRDENILKQIFGILKAPFRDK    529
XP_038409684.1             RFVIQLLEDLVFFVSDVPNNGQNVLDIMVTKPNRERQKLMREQNILKQIFGILKAPFRDK    540
NP_002215.2                RFVIQLLEDLVFFVSDVPNNGQNVLDIMVTKPNRERQKLMREQNILKQVFGILKAPFREK    529
                           *****************************************::*****:*********:*

sp|P70227|ITPR3_MOUSE      GGEGPLVRLEELSDQKNAPYQYMFRLCYRVLRHSQEDYRKNQEHIAKQFGMMQSQIGYDI    589
sp|Q63269|ITPR3_RAT        GGEGPLVRLEELSDQKNAPYQYMFRLCYRVLRHSQEDYRKNQEHIAKQFGMMQSQIGYDI    589
XP_038409684.1             GGEGPLVRLEELSDQKNAPYQHMFRLCYRVLRHSQEDYRKNQEHIAKQFGMMQSQIGYDI    600
NP_002215.2                GGEGPLVRLEELSDQKNAPYQHMFRLCYRVLRHSQEDYRKNQEHIAKQFGMMQSQIGYDI    589
                           *********************:**************************************

sp|P70227|ITPR3_MOUSE      LAEDTITALLHNNRKLLEKHITKTEVETFVSLVRKNREPRFLDYLSDLCVSNRIAIPVTQ    649
sp|Q63269|ITPR3_RAT        LAEDTITALLHNNRKLLEKHITKTEVETFVSLVRKNREPRFLDYLSDLCVSNRIAIPVTQ    649
XP_038409684.1             LAEDTITALLHNNRKLLEKHITKTEVETFVSLVRKNREPRFLDYLSDLCVSNHIAIAVTQ    660
NP_002215.2                LAEDTITALLHNNRKLLEKHITKTEVETFVSLVRKNREPRFLDYLSDLCVSNHIAIPVTQ    649
                           ****************************************************:*** ***

sp|P70227|ITPR3_MOUSE      ELICKCVLDPKNSDILIQTELRPVKEMAQSHEYLSIEYSEEEVWLTWTDRNNEHHEKSVR    709
sp|Q63269|ITPR3_RAT        ELICKCVLDPKNSDILIQTELRPVKEMAQSHEYLSIEYSEEEVWLTWTDRNNEHHEKSVR    709
XP_038409684.1             ELICKCVLDPKNSDILIQTELRPVKEMAQSHEYLSIEYSEEEVWLTWTDKNNEHHEKSVR    720
NP_002215.2                ELICKCVLDPKNSDILIRTELRPVKEMAQSHEYLSIEYSEEEVWLTWTDKNNEHHEKSVR    709
                           *****************:*******************************:**********

sp|P70227|ITPR3_MOUSE      QLAQEARAGNAHDENVLSYYRYQLKLFARMCLDRQYLAIDEISKQLGVELLFLCMADEML    769
sp|Q63269|ITPR3_RAT        QLAQEARAGNAHDENVLSYYRYQLKLFARMCLDRQYLAIDEISKQLGVELLFLCMADEML    769
XP_038409684.1             QLAQEARAGNAHDENVLSYYRYQLKLFARMCLDRQYLAIDEISQQLGVDLIFLCMADEML    780
NP_002215.2                QLAQEARAGNAHDENVLSYYRYQLKLFARMCLDRQYLAIDEISQQLGVDLIFLCMADEML    769
                           *******************************************:****:*:*********

sp|P70227|ITPR3_MOUSE      PFDLRASFCHLMLHVHVDRDPQELVTPVKFARLWTEIPTAITIKDYDSNLNASRDDKKNK    829
sp|Q63269|ITPR3_RAT        PFDLRASFCHLMLHVHVDRDPQELVTPVKFARLWTEIPTAITIKDYDSNLNASRDDKKNK    829
XP_038409684.1             PFDLRASFCHLMLHVHVDRDPQELVTPVKFARLWTEIPTAITIKDYDSNLNASRDDKKNK    840
NP_002215.2                PFDLRASFCHLMLHVHVDRDPQELVTPVKFARLWTEIPTAITIKDYDSNLNASRDDKKNK    829
                           ************************************************************

sp|P70227|ITPR3_MOUSE      FASTMEFVEDYLNNVVSEAVPFANDEKNILTFEVVSLAHNLIYFGFYSFSELLRLTRTLL    889
sp|Q63269|ITPR3_RAT        FASTMEFVEDYLNNVVGEAVPFANDEKNILTFEVVSLAHNLIYFGFYSFSELLRLTRTLL    889
XP_038409684.1             FASTMDFVEDYLNNVVSEAVPFANEEKNKLTFEVVSLAHNLIYFGFYSFSELLRLTRTLL    900
NP_002215.2                FANTMEFVEDYLNNVVSEAVPFANEEKNKLTFEVVSLAHNLIYFGFYSFSELLRLTRTLL    889
                           **.**:**********.*******:*** *******************************
```

```
sp|P70227|ITPR3_MOUSE      GIIDCIQAPAAMLQAYEEPGGKNVRRSIQGVGHMMSTMVLSRKQSVFGASSLPAGVGVPE    949
sp|Q63269|ITPR3_RAT        GIIDCIQAPAAVLQAYEEPGGKNVRRSIQGVGHMMSTMVLSRKQSVFGASSLPTGVGVPE    949
XP_038409684.1             GIIDCVQGPPAMLQAYDDSGGKNVRRSIQGVGHMMSTMVLSRKQSVFGAPSLSGGTGATE    960
NP_002215.2                GIIDCVQGPPAMLQAYEDPGGKNVRRSIQGVGHMMSTMVLSRKQSVFSAPSLSAGASAAE    949
                           *****:*.* *:****:: ****************************.* **  *... *

sp|P70227|ITPR3_MOUSE      QLDRSKFEDNEHTVVMETKLKILEILQFILNVRLDYRISYLLSVFKKEFVEVFPMQDSGA    1009
sp|Q63269|ITPR3_RAT        QLDRSKFEDNEHTVVMETKLKILEILQFILNVRLDYRISYLLSVFKKEFVEVFPMQDSGA    1009
XP_038409684.1             PLDRSKFENNEDIVVMETKLKILEILQFILNVRLDYRISYLLSVFKKEFVEVFPMQDSGA    1020
NP_002215.2                PLDRSKFEENEDIVVMETKLKILEILQFILNVRLDYRISYLLSVFKKEFVEVFPMQDSGA    1009
                            *******:**. ***********************************************

sp|P70227|ITPR3_MOUSE      DGTAPAFDSSTATMNLDRIGEQAEAMFGVGKTSSMLEVDDEGGRMFLRVLLHLTMHDYPS    1069
sp|Q63269|ITPR3_RAT        DGTAPAFDSSTANMNLDRIGEQAEAMFGVGKTSSMLEVDDEGGRMFLRVLLHLTMHDYPP    1069
XP_038409684.1             DGTAPAFDSTTANMNLDRIGEQAEAMFGVGKTSSMLEVDDEGGRTFLRVLIHLTMHDYAP    1080
NP_002215.2                DGTAPAFDSTTANMNLDRIGEQAEAMFGVGKTSSMLEVDDEGGRMFLRVLIHLTMHDYAP    1069
                           *********:**.******************************* *****:*******

sp|P70227|ITPR3_MOUSE      LVSGALQLLFKHFSQRQEAMHTFKQVQLLISAQDVENYKVIKSELDRLRTMVEKSELWVD    1129
sp|Q63269|ITPR3_RAT        LVSGALQLLFKHFSQRQEAMHTFKQVQLLISAQDVENYKVIKSELDRLRTMVEKSELWVD    1129
XP_038409684.1             LVSGALQLLFKHFSQRQEAMHTFKQVQLLISAQDVENYKVIKSELDRLRTMVEKSELWVD    1140
NP_002215.2                LVSGALQLLFKHFSQRQEAMHTFKQVQLLISAQDVENYKVIKSELDRLRTMVEKSELWVD    1129
                           ************************************************************

sp|P70227|ITPR3_MOUSE      KKGSVKGEEVEAGATKDKKERPSDEEGFLQPHGEKSSENYQIVKGILERLNKMCGVGEQM    1189
sp|Q63269|ITPR3_RAT        KKGSVKGEEGEAGASKDKKERPSDEEGFLQPHGEKSSENYQIVKGILERLNKMCGVGEQM    1189
XP_038409684.1             KKGSGKGEEVDTGAAKDKKERPTDEEGFLHPPGEKSSENYQIVKGILERLNKMCGVGEQM    1200
NP_002215.2                KKGSGKGEEVEAGAAKDKKERPTDEEGFLHPPGEKSSENYQIVKGILERLNKMCGVGEQM    1189
                           **** **** ::**:*******:******:* ****************************

sp|P70227|ITPR3_MOUSE      RKKQQRLLKNMDAHKVMLDLLQIPYDKSDNKMLEILRYTHQFLQKFCAGNPGNQALLHKH    1249
sp|Q63269|ITPR3_RAT        RKKQQRLLKNMDAHKVMLDLLQIPYDKNDNKMMEILRYTHQFLQKFCAGNPGNQALLHKH    1249
XP_038409684.1             RKKQQRLLKNMDAHKVMLDLLQIPYDKGDAKMMEILRYTHQFLQKFCAGNPGNQALLHKH    1260
NP_002215.2                RKKQQRLLKNMDAHKVMLDLLQIPYDKGDAKMMEILRYTHQFLQKFCAGNPGNQALLHKH    1249
                           ***************************.* **:***************************

sp|P70227|ITPR3_MOUSE      LQLFLTPGLLEAETMQHIFLNNYQLCSEISEPVLQHFVHLLATHGRHVQYLDFLHTVIKA    1309
sp|Q63269|ITPR3_RAT        LQLFLTPGLLEAETMQHIFLNNYQLCSEISEPVLQHFVHCWPTHGRHVQYLDFLHTVIKA    1309
XP_038409684.1             LHLFLTPGLLEAETMQHIFLNNYQLCSEISEPVLQHFVHLLATHGRHVQYLDFLHTVIKA    1320
NP_002215.2                LHLFLTPGLLEAETMQHIFLNNYQLCSEISEPVLQHFVHLLATHGRHVQYLDFLHTVIKA    1309
                           *:*************************************   ******************

sp|P70227|ITPR3_MOUSE      EGKYVKKCQDMIMTELTNAGDDVVVFYNDKASLAHLLDMMKAARDGVEDHSPLMYHISLV    1369
sp|Q63269|ITPR3_RAT        EGKYVKKCQDMIMTELTNAGDDVVVFYNDKASLAHLLDMMKAARDGVEDHSPLMYHISLV    1369
XP_038409684.1             EGKYVKKCQDMIMTELTNAGDDVVVFYNDKASLAHLLDMMKAARDGVEDHSPLMYHISLV    1380
NP_002215.2                EGKYVKKCQDMIMTELTNAGDDVVVFYNDKASLAHLLDMMKAARDGVEDHSPLMYHISLV    1369
                           ************************************************************

sp|P70227|ITPR3_MOUSE      DLLAACAEGKNVYTEIKCTSLLPLEDVVTVVTHEDCITE--------VKMAYVNFVNHCY    1421
sp|Q63269|ITPR3_RAT        DLLAACAEGKNVYTEIKCTSLLPLEDVVSVVTHEDCITE--------VKMAYVNFVNHCY    1421
XP_038409684.1             DLLAACAEGKNVYTEIKCTSLLPLEDVVSVVTHEDCITEVFWGLGEVVKMAYVNFVNHCY    1440
NP_002215.2                DLLAACAEGKNVYTEIKCTSLLPLEDVVSVVTHEDCITE--------VKMAYVNFVNHCY    1421
                           ****************************:**********        *************

sp|P70227|ITPR3_MOUSE      VDTEVEMKEIYTSNHIWTLFENFTLDMALVCNKREKRLSDPTLEKYVLTVVLDTISAFFS    1481
sp|Q63269|ITPR3_RAT        VDTEVEMKEIYTSNHIWTLFENFTLDMALVCNKREKRLSDPTLEKYVLTVVLDTISAFFS    1481
XP_038409684.1             VDTEVEMKEIYTSNHIWTLFENFTLDMARVCSKREKRLADPTLEKYVLTVVLDTINAFFS    1500
NP_002215.2                VDTEVEMKEIYTSNHIWTLFENFTLDMARVCSKREKRVADPTLEKYVLSVVLDTINAFFS    1481
                           **************************** **.*****::*********:******.****

sp|P70227|ITPR3_MOUSE      SPFSENSTSLQTHQTIVVQLLQSTTRLLECPWLQQQHKGSVEACVRTLAMVAKSRAILLP    1541
sp|Q63269|ITPR3_RAT        SPFSENSTSLQTHQTIVVQLLQSTTRLLECPWLQQQHKGSVEACVRTLAMVAKSRAILLP    1541
XP_038409684.1             SPFSENSTSLQTHQTIVVQLLQSTTRLLECPWLQQQHKGSVEACIRTLAMVAKGRAISLP    1560
NP_002215.2                SPFSENSTSLQTHQTIVVQLLQSTTRLLECPWLQQQHKGSVEACIRTLAMVAKGRAILLP    1541
                           ********************************************:********.*** **

sp|P70227|ITPR3_MOUSE      MDLDAHMSALLSSGGSCSAAAQRSAANYKTATRTFPRVIPTANQWDYKNIIEKLQDIIMA    1601
sp|Q63269|ITPR3_RAT        MDLDAHMSALLSSGGSCSAAAQRSAANYKTATRTFPRVIPTANQWDYKNIIEKLQDIITA    1601
XP_038409684.1             MDLDAHISSLLSSSASCVAAAQRNASNYKAATRAFPRVTPTANQWDYKNIIEKLQDIITA    1620
NP_002215.2                MDLDAHISSMLSSGASCAAAAQRNASSYKATTRAFPRVTPTANQWDYKNIIEKLQDIITA    1601
                           ******:*::***..** *****.*:.**::**:**** ******************* *

sp|P70227|ITPR3_MOUSE      LEERLKPLVQAELSVLVDMLHWPELLFPEGSEAYQRCESGGFLSKLIRHTKGLMESEEKL    1661
sp|Q63269|ITPR3_RAT        LEERLKPLVQAELSVLVDMLHWPELLFLEGSEAYQRCESGGFLSKLIRHTKGLMESEEKL    1661
XP_038409684.1             LEERLRPLVQAELSVLVDVLHWPELLFLEGSEAYQRCESGGFLSKLIQHTKDLMESEEKL    1680
NP_002215.2                LEERLKPLVQAELSVLVDVLHWPELLFLEGSEAYQRCESGGFLSKLIQHTKDLMESEEKL    1661
                           *****:************:******** *******************:***.********

sp|P70227|ITPR3_MOUSE      CVKVLRTLQQMLLKKSKFGDRGNQLRKMLLQNYLQNRKSGARGELTDPTGSGLDQDWSAI    1721
sp|Q63269|ITPR3_RAT        CVKVLRTLQQMLQKKSKYGDRGNQLRKMLLQNYLQNRKSGPRGELTDPTGSGVDQDWSAI    1721
XP_038409684.1             CVKVLRTLQQMLLKKAKYGDRGNQLRKMLLQNYLQNRKSSSRGDLPDPMGTGLDQDWSAI    1740
NP_002215.2                CIKVLRTLQQMLLKKTKYGDRGNQLRKMLLQNYLQNRKSTSRGDLPDPIGTGLDPDWSAI    1721
                           *:********** **:*:*********************  **:* ** *:*:* *****

sp|P70227|ITPR3_MOUSE      AATQCRLDKEGATKLVCDLITSTKNEKIFQESIGLAIRLLDGGNTEIQKSFYNLMTSDKK    1781
sp|Q63269|ITPR3_RAT        AATQCRLDKEGATKLVCDLITSTKNEKIFQESIGLAIRLLDGGNTEIQKSFYNLMTSDKK    1781
XP_038409684.1             AATQCRLDKEGATKLVCDLITSTKNEKIFQESIGLAIRLLDGGNTEIQKSFYNLMMTDKK    1800
NP_002215.2                AATQCRLDKEGATKLVCDLITSTKNEKIFQESIGLAIHLLDGGNTEIQKSFHNLMMSDKK    1781
                           *************************************:*************:*** :***

sp|P70227|ITPR3_MOUSE      SERFFKVLHDRMKRAQQETKSTVAVNMSDLGSQPREDREPADPATKGRVSSFSMPSS-SR    1840
sp|Q63269|ITPR3_RAT        SERFFKVLHDRMKRAQQETKSTVAVNMSDLGSQPREDREPADPTTKGRVSSFSMPSS-SR    1840
XP_038409684.1             SERFFKVLHDRMKRAQQETKSTVAVNMNDLGSQPREDREPAGPTTKGRVASFSMPGSPSR    1860
NP_002215.2                SERFFKVLHDRMKRAQQETKSTVAVNMNDLGSQPHEDREPVDPTTKGRVASFSIPGSSSR    1841
                           ***************************.******:*****..*:*****:***:*.* **

sp|P70227|ITPR3_MOUSE      YLLGLGLHRGHDMSERAQNNEMGTSVLIMRPILRFLQLLCENHNRDLQNFLRCQNNKTNY    1900
sp|Q63269|ITPR3_RAT        YSLGPGLHRGHDVSERAQNNEMGTSVLIMRPILRFLQLLCENHNRDLQNFLRCQNNKTNY    1900
XP_038409684.1             YSLGPSLRRGHEVGERVQSNEMGMSVLIMQPILRFLQLLCENHNRDLQNFLRSQNNKTNY    1920
NP_002215.2                YSLGPSLRRGHEVSERVQSSEMGTSVLIMQPILRFLQLLCENHNRDLQNFLRCQNNKTNY    1901
                           * ** .*:***::.**.*..*** *****:**********************.*******
```

```
sp|P70227|ITPR3_MOUSE         NLVCETLQFLDIMCGSTTGGLGLLGLYINEDNVGLVIQTLETLTEYCQGPCHENQTCIVT   1960
sp|Q63269|ITPR3_RAT           NLVCETLQFLDIMCGSTTGGLGLLGLYINEDNVGLVIQTLETLTEYCQGPCHENQTCIVT   1960
XP_038409684.1                NLVCETLQFLDIMCGSTTGGLGLLGLYINEDNVGLVIQTLETLTEYCQGPCHENQTCIVT   1980
NP_002215.2                   NLVCETLQFLDIMCGSTTGGLGLLGLYINEDNVGLVIQTLETLTEYCQGPCHENQTCIVT   1961
                              ************************************************************

sp|P70227|ITPR3_MOUSE         HESNGIDIITALILNDISPLCKYRMDLVLQLKDNASKLLLALMESRHDSENAERILISLR   2020
sp|Q63269|ITPR3_RAT           HESNGIDIITALILNDISPLCKYRMDLVLQLKDNASKLLLALMESRHDSENAERILISLR   2020
XP_038409684.1                HESNGIDIITALILNDISPLCKYRMDLVLQLKDNASKLLLALMESRHDSENAERILISLR   2040
NP_002215.2                   HESNGIDIITALILNDISPLCKYRMDLVLQLKDNASKLLLALMESRHDSENAERILISLR   2021
                              ************************************************************

sp|P70227|ITPR3_MOUSE         PQELVDVIKKAYLQEEERENSEVSPREVGHNIYILALQLSRHNKQLQHLLKPVRRIQEEE   2080
sp|Q63269|ITPR3_RAT           PQELVDVIKKAYLQEEERENSEVSPREVGHNIYILALQLSRHNKQLQHLLKPVKRIQEEE   2080
XP_038409684.1                PQELVDVIKKAYLQEDERENSEVSPREVGHNIYILALQLSRHNKQLQHLLKPVRRIQEEE   2100
NP_002215.2                   PQELVDVIKKAYLQEEERENSEVSPREVGHNIYILALQLSRHNKQLQHLLKPVKRIQEEE   2081
                              ***************:*************************************:******

sp|P70227|ITPR3_MOUSE         AEGISSMLSLNNKQLSQMLKSSAPAQEEEEDPLAYYENHTSQIEIVRQDRSMEQIVFPVP   2140
sp|Q63269|ITPR3_RAT           AEGISSMLSLNNKQLSQMLKSSAPAQEEEEDPLAYYENHTSQIEIVRQDRSMEQIVFPVP   2140
XP_038409684.1                AEGISSMLSLNNKQLSQMLKSSAPAQEEEEDPLAYYENHTSQIEIVRQDRSMEQIVFPVP   2160
NP_002215.2                   AEGISSMLSLNNKQLSQMLKSSAPAQEEEEDPLAYYENHTSQIEIVRQDRSMEQIVFPVP   2141
                              ************************************************************

sp|P70227|ITPR3_MOUSE         AICQFLTEETKHRLFTTTEQDEQGSKVSDFFDQSSFLHNEMEWQRRLRSMPLIYWFSRRM   2200
sp|Q63269|ITPR3_RAT           AICQFLTEETKHRLFTTTEQDEQGSKVSDFFDQSSFLHNEMEWQRRLRSMPLIYWFSRRM   2200
XP_038409684.1                GICQFLTEETKHRLFTTTEQDEQGSKVSDFFDQSSFLHNEMEWQRKLRSMPLIYWFSRRM   2220
NP_002215.2                   GICQFLTEETKHRLFTTTEQDEQGSKVSDFFDQSSFLHNEMEWQRKLRSMPLIYWFSRRM   2201
                              .********************************************:**************

sp|P70227|ITPR3_MOUSE         TLWGSISFNLAVFINIIIAFFYPYVEGASTGVLGSPLISLLFWILICFSIAALFTKRYSV   2260
sp|Q63269|ITPR3_RAT           TLWGSISFNLAVFINIIIAFFYPYVEGASTGVLGSPLISLLFWILICFSIAALFTKHYSV   2260
XP_038409684.1                TLWGSISFNLAVFINIIIAFFYPYMEGASTGVLGSPLISLLFWILICFSIVALFTKRYSI   2280
NP_002215.2                   TLWGSISFNLAVFINIIIAFFYPYMEGASTGVLDSPLISLLFWILICFSIAALFTKRYSI   2261
                              ************************:********.****************.*****:**:

sp|P70227|ITPR3_MOUSE         RPLIVALILRSIYYLGIGPTLNILGALNLTNKIVFVVSFVGNRGTFIRGYKAMVMDMEFL   2320
sp|Q63269|ITPR3_RAT           RPLIVALVLRSIYYLGIGPTLNILGALNLTNKIVFVVSFVGNRGTFIRGYKAMVMDMEFL   2320
XP_038409684.1                RPLIVALILRSIYYLGIGPTLNILGALNLTNKIVFVVSFVGNRGTFIRGYKAMVMDVEFL   2340
NP_002215.2                   RPLIVALILRSIYYLGIGPTLNILGALNLTNKIVFVVSFVGNRGTFIRGYKAMVMDMEFL   2321
                              *******:************************************************:***

sp|P70227|ITPR3_MOUSE         YHVGYILTSVLGLFAHELFYSILLFDLIYREETLFNVIKSVTRNGRSILLTALLALILVY   2380
sp|Q63269|ITPR3_RAT           YHVGYILTSVLGLFAHELFYSILLFDLIYREETLFNVIKSVTRNGRSILLTALLALILVY   2380
XP_038409684.1                YHVGYILTSVLGLFVHELFYSILLFDLIYREETLFNVIKSVTRNGRSILLTALLALILVY   2400
NP_002215.2                   YHVGYILTSVLGLFAHELFYSILLFDLIYREETLFNVIKSVTRNGRSILLTALLALILVY   2381
                              **************.*********************************************

sp|P70227|ITPR3_MOUSE         LFSIVGFLFLKDDFILEVDRLPGNHSRASPLGMPHGAATFMGTCSGDKMDCVSEVSVPEI   2440
sp|Q63269|ITPR3_RAT           LFSIVGFLFLKDDFILEVDRLPGNHSRASTLGMPHGAATFMGTCSGDKMDCVSEVSVPEI   2440
XP_038409684.1                LFSIVGFLFLKDDFILEVDRLPGNHSGASPLGMPHGAATFVGTCSGDQLNCDSGVSVPEV   2460
NP_002215.2                   LFSIVGFLFLKDDFILEVDRLPNNHSTASPLGMPHGAAAFVDTCSGDKMDCVSGLSVPEV   2441
                              **********************.*** ** ********:*:.*****:::* * :****:

sp|P70227|ITPR3_MOUSE         LEEDEEPDSTERACDTLLMCIVTVMNHGLRNGGGVGDILRKPSKDESLFPARVVYDLLFF   2500
sp|Q63269|ITPR3_RAT           LEEDEELDSTERACDTLLMCIVTVMNHGLRNGGGVGDILRKPSKDESLFPARVVYDLLFF   2500
XP_038409684.1                LEGNEELESTERACDTLLMCIVTVMNHGLRNGGGVGDILRKPSKDESLFPARVVYDLLFF   2520
NP_002215.2                   LEEDRELDSTERACDTLLMCIVTVMNHGLRNGGGVGDILRKPSKDESLFPARVVYDLLFF   2501
                              ** :.* :****************************************************

sp|P70227|ITPR3_MOUSE         FIVIIIVLNLIFGVIIDTFADLRSEKQKKEEILKTTCFICGLERDKFDNKTVSFEEHIKL   2560
sp|Q63269|ITPR3_RAT           FIVIIIVLNLIFGVIIDTFADLRSEKQKKEEILKTTCFICGLERDKFDNKTVSFEEHIKL   2560
XP_038409684.1                FIVIIIVLNLIFGVIIDTFADLRSEKQKKEEILKTTCFICGLERDKFDNKTVSFEEHIKF   2580
NP_002215.2                   FIVIIIVLNLIFGVIIDTFADLRSEKQKKEEILKTTCFICGLERDKFDNKTVSFEEHIKL   2561
                              ***********************************************************:

sp|P70227|ITPR3_MOUSE         EHNMWNYLYFIVLVRVKNKTDYTGPESYVAQMIKNKNLDWFPRMRAMSLVSGEGEGEQNE   2620
sp|Q63269|ITPR3_RAT           EHNMWNYLYFIVLVRVKNKTDYTGPESYVAQMIKNKNLDWFPRMRAMSLVSGEGEGEQNE   2620
XP_038409684.1                EHNMWNYLYFIVLVRVKNKTDYTGPESYVAQMIKNKNLDWFPRMRAMSLVSNEGEGEQNE   2640
NP_002215.2                   EHNMWNYLYFIVLVRVKNKTDYTGPESYVAQMIKNKNLDWFPRMRAMSLVSNEGEGEQNE   2621
                              ***************************************************.********

sp|P70227|ITPR3_MOUSE         IRILQEKLGSTMKLVSHLTSQLNELKEQMTEQRKRRQLGFVDVQNCMSR              2670
sp|Q63269|ITPR3_RAT           IRILQEKLGSTMKLVSHLTAQLNELKEQMTEQRKRRQLGFVDVQNCMSR              2670
XP_038409684.1                IRILQDKLNSTMKLVSHLTAQLNELKEQMTEQRKRRQLGFVDVQNCMSR              2690
NP_002215.2                   IRILQDKLNSTMKLVSHLTAQLNELKEQMTEQRKRRQLGFVDVQNCISR              2671
                              *****:**.**********:**************************:**
```
