# Supplementary figures and images for: IP3 receptor depletion in a spontaneous canine model of Charcot-Marie-Tooth disease 1J with amelogenesis imperfecta
Source: PLoS Genet. 2025 Jan 13;21(1):e1011328. doi: 10.1371/journal.pgen.1011328 (PMC11761660; doi:10.1371/journal.pgen.1011328)

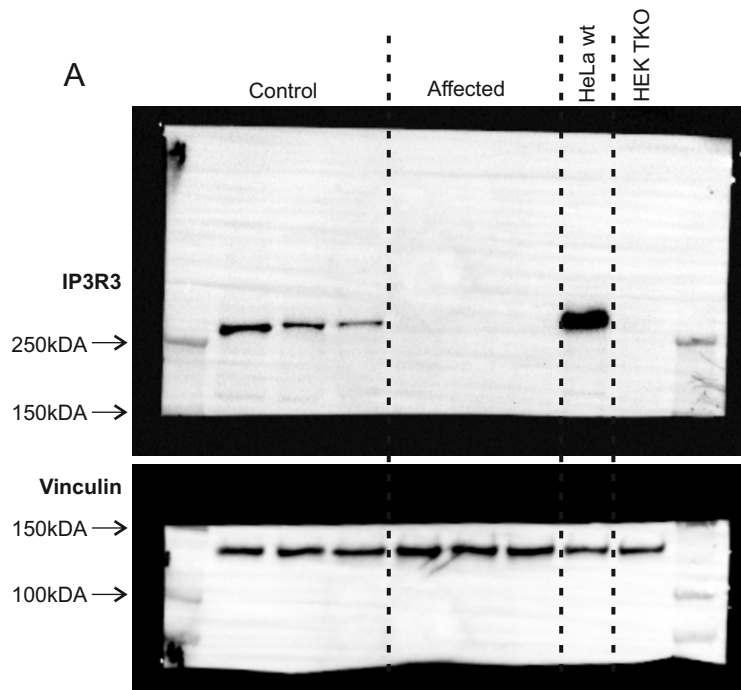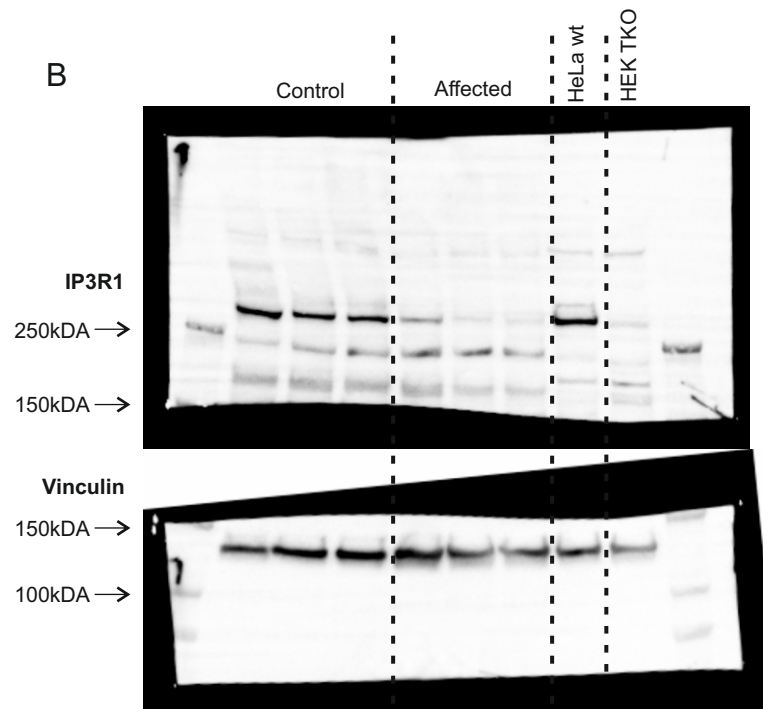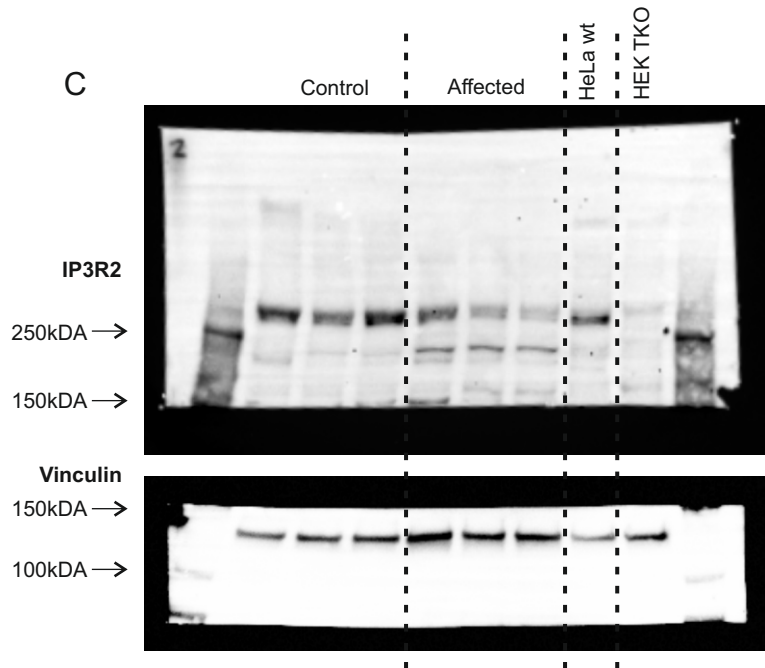

Supplement: S2 Fig — (PDF) [file pgen.1011328.s002.pdf]

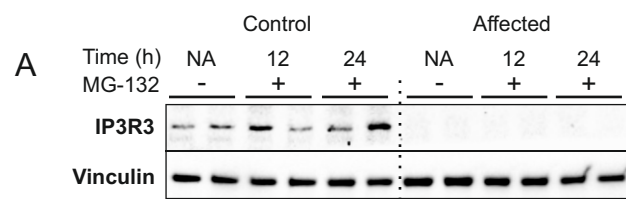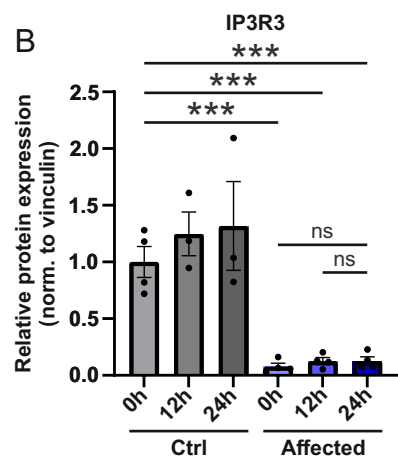

Supplement: S3 Fig — (PDF) [file pgen.1011328.s003.pdf]

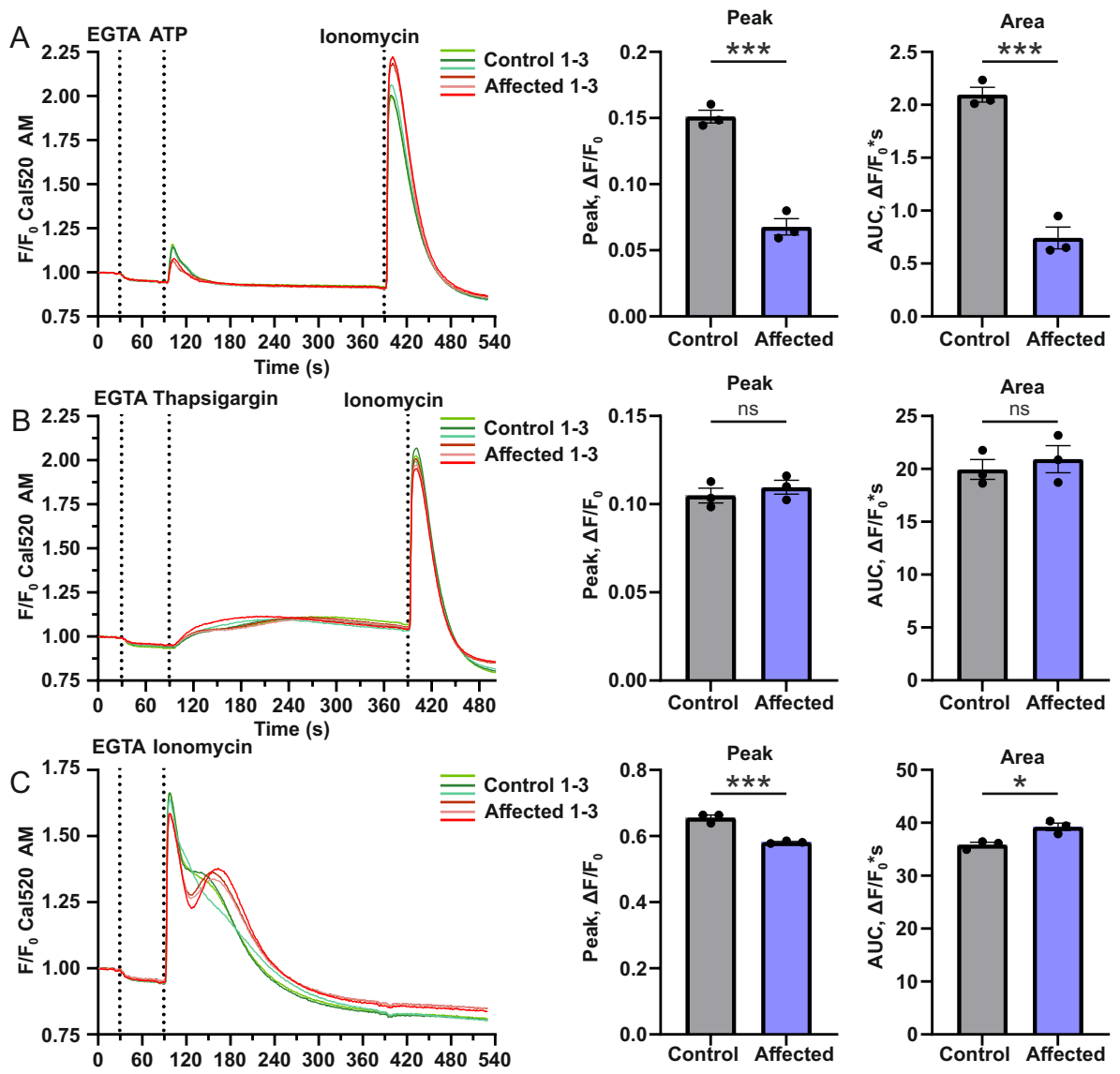

Supplement: S4 Fig — (PDF) [file pgen.1011328.s004.pdf]
