# Supplementary material for: IP3 receptor depletion in a spontaneous canine model of Charcot-Marie-Tooth disease 1J with amelogenesis imperfecta
Source: PLoS Genet. 2025 Jan 13;21(1):e1011328. doi: 10.1371/journal.pgen.1011328 (PMC11761660; doi:10.1371/journal.pgen.1011328)
Supplement: S2 Table — (PDF) [file pgen.1011328.s006.pdf]

Supplementary table 2. Electromyographic findings of the affected dogs.

Lancashire Heeler	Ulnar (normal >60 m/s)	Ishiadicus/peroneal (normal >80 m/s)	Fibrillation potentials	Positive sharp waves	CMAP amplitude	CMAP duration
Affected 1 (age 10y 5m)	58 m/s	62 m/s	+	+	↓	↑
Affected 2 (6y 10m)	44 m/s	76 m/s	+	+	↓	↑
Affected 3 (6y 5m & 7y 6m)	80 m/s	95 m/s	+	+	-	-
