# Supplementary material for: IP3 receptor depletion in a spontaneous canine model of Charcot-Marie-Tooth disease 1J with amelogenesis imperfecta
Source: PLoS Genet. 2025 Jan 13;21(1):e1011328. doi: 10.1371/journal.pgen.1011328 (PMC11761660; doi:10.1371/journal.pgen.1011328)
Supplement: S7 Table — (PDF) [file pgen.1011328.s011.pdf]

Supplementary table 7. Values used for parameter optimization and detection of shared ROH in PLINK.

	values by analysis type	
parameter	parameter optimization	detection of shared ROH
--homozyg-window-snp	20	20
--homozyg-window-missing	1	1
--homozyg-window-het	0	1
--homozyg-window-threshold	0.05	0.05
--homozyg-snp	70	54
--homozyg-kb	1000 kb	1000 kb
--homozyg-gap	2000 kb (when unvaried)<br><br>20-1000 kb (when varied)	200 kb
--homozyg-density	200 kb/snp (when unvaried)<br><br>10-125 kb/snp (when varied)	25 kb/snp
