# Supplementary material for: IP3 receptor depletion in a spontaneous canine model of Charcot-Marie-Tooth disease 1J with amelogenesis imperfecta
Source: PLoS Genet. 2025 Jan 13;21(1):e1011328. doi: 10.1371/journal.pgen.1011328 (PMC11761660; doi:10.1371/journal.pgen.1011328)
Supplement: S8 Table — (PDF) [file pgen.1011328.s012.pdf]

Supplementary table 8. Primers used in the study

Oligonucleotide	Sequence (5'->3')	Application	Locus & amplicon size
canine ITPR3-Fw	CCTGTCCAAGTGAGCGAGAT	PCR & Sanger sequencing	Exon 36, 311 bp
canine ITPR3-Rv	CGTACTTGGCCTTCTTGAGC	PCR & Sanger sequencing	Exon 37
canine ITPR1-qPCR-Fw	GTCCCAAGAAACTCCTATGTCC	qRT-PCR	Exon 14/15, 97 bp
canine ITPR1-qPCR-Rv	CGGGTTTCTCTTCTTCCTTGT	qRT-PCR	Exon 15
canine ITPR2-qPCR-Fw	GAGAAGCGAGGGTGACAATATC	qRT-PCR	Exon 5/6, 91 bp
canine ITPR2-qPCR-Rv	CTCTATGTTGCTGGCATGTAGT	qRT-PCR	Exon 6
canine ITPR3-qPCR-Fw	CCATCGTGTCTGTTCCTGTATC	qRT-PCR	Exon 13, 129bp
canine ITPR3-qPCR-Rv	TGACAAACCTGCGATCATTCT	qRT-PCR	Exon 13/14
canine GAPDH-qPCR-Fw	CTGGGGCTCACTTGAAAGG	qRT-PCR	Exon 1, 72 bp
canine GAPDH-qPCR-Rv	CAAACATGGGGGCATCAG	qRT-PCR	Exon 1
